# Composite Hole-Transporting Materials Based on 9,10-Dimethoxyphenanthrene Cores and Spiro-OMeTAD for Efficient and Stable Perovskite Solar Cells

**DOI:** 10.1021/acsomega.5c01513

**Published:** 2025-05-14

**Authors:** Jijitha Vailassery, Gebremariam Zebene Wubie, Jia-Wei She, Wen-Ti Wu, Hsiao-hua Yu, Shih-Sheng Sun

**Affiliations:** † Institute of Chemistry, 38017 Academia Sinica, No. 128, Academia Road, Sec. 2, Nankang, Taipei 115, Taiwan, R. O. C; ‡ Taiwan International Graduate Program, Sustainable Chemical Science and Technology, Hsinchu 300, Taiwan, R. O. C; § Department of Applied Chemistry, National Yang Ming Chiao Tung University, Hsinchu 300, Taiwan, R. O. C

## Abstract

The hole transport material (HTM) in perovskite solar cells (PSCs) is a critical component due to its profound influence on the hole extraction, surface passivation, shielding the perovskite from moisture, and oxygen directly impacting on the overall performance and stability of the devices. The widely used HTM, spiro-OMeTAD (2,2′,7,7′-tetrakis­(*N*,*N*-di-*p*-methoxyphenyl-amine)­9,9′-spirofluorene), for n-i-p PSCs suffers from low conductivity and poor hole mobility in its pristine form. In this work, we designed two structurally simple and cost-effective isomeric small molecules (2,7-OPOT and 3,6-OPOT), featuring a 9,10-dimethoxyphenanthrene core in a D-π-D structure, and mixed them with spiro-OMeTAD to form composite HTMs, S-2,7-OPOT, and S-3,6-OPOT. The champion device with S-3,6-OPOT-based composite HTM attained a power conversion efficiency (PCE) of 18.8% (*J*_sc_ = 23.9 mA cm^–2^, *V*_oc_ = 1.05 V, and FF = 74.92%), outperforming devices based on S-2,7-OPOT (18.6%) and pristine spiro-OMeTAD (17.7%). The S-3,6-OPOT-based PSC also displayed superior durability, retaining over 81% of its initial PCE after 60 days of ambient storage condition without encapsulation. These findings confirm that systematic mixing of organic small molecules with spiro-OMeTAD is a promising approach to improve the photovoltaic performance and durability of PSCs, even with reduced dopant loading in spiro-OMeTAD.

## Graphical abstract

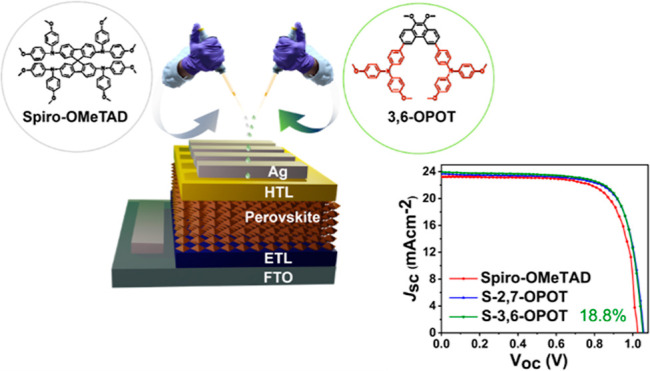

## Introduction

Organic–inorganic hybrid perovskite solar cells (PSCs) have made remarkable progress as an emerging third-generation photovoltaic technology because of their charming opto-electrical properties, such as high absorption coefficients, extended carrier diffusion lengths, as well as low exciton binding energies.^1–4^ Within just 1.5 decades, the power conversion efficiency (PCE) of PSCs has surpassed 26%, making an unprecedented advancement in the photovoltaics sector.^5,6^ Among the main functional components of PSCs, hole-transporting materials (HTMs) serve crucial roles in facilitating hole extraction, reducing charge recombination, passivating surface defects, and shielding the perovskite layer from moisture and oxygen. These functions ultimately influence the PCEs and stability of the devices.^7–12^ HTMs with diverse molecular architectures have been developed to enhance the photovoltaic performance and durability of PSC devices.^13–19^ In this regard, 2,2′,7,7′-tetrakis­(*N*,*N*-di-*p*-methoxyphenyl-amine)­9,9′-spirofluorene (spiro-OMeTAD) remains the most widely explored hole transport material (HTM) in the n-i-p device architecture of PSCs.^20–23^ However, pristine spiro-OMeTAD suffers from low hole mobility and poor conductivity,^24^ necessitating the employing of dopants and additives, including lithium bis­(trifluoromethanesulfonyl)­imide (LiTFSI) and 4-*tert*-butylpyridine (tBP), to improve conductivity and facilitate dopant dispersion in the spiro-OMeTAD organic matrix.^25–27^ The addition of dopants usually lowers the glass transition temperatures (*T*_g_) of HTMs, leading to aggregation of materials under thermal stress. The volatilization of the tBP further degrades the film quality,^28,29^ while the hygroscopic nature of LiTFSI accelerates perovskite decomposition, adversely affecting device stability.^30–32^

Additionally, perovskites often require surface treatments with aryl or alkylammonium iodides to address energy level mismatch between perovskite and spiro-OMeTAD as well as for the surface passivation.^33,34^ However, these reagents can react with PbI_2_, leading to interstitial vacancies and defects.^35–37^ Recent studies indicate that defects present at the surface of solution-processed perovskite films and interface of perovskite/HTM serve as centers for charge carrier recombination that inhibits efficient charge transfer from the perovskite layer to the HTM.^38,39^ In addition, surface defects such as under-coordinated lead can expedite the degradation of PSCs under prolonged illumination.^40,41^ These scenarios could have a substantial effect on the PSC device’s stability and performance, presenting a big barrier to mass production as well as commercialization. The introduction of interfacial layers has been proposed as a potential solution to address this issue, particularly in p-i-n devices.^42–44^ However, the necessity for thin interlayers to facilitate tunneling through them complicates the fabrication process.^45^ The design of multifunctional HTMs, which act as defect passivators, interfacial charge transporters, and protective layers, is an effective strategy for n-i-p type PSCs. Amalgamation of heteroatoms like N, O, S, Se, and F in an HTM structure could be beneficial to passivate the surface trap state of perovskite films.^46–51^ The surface passivation as well as reduced carrier recombination through these HTMs has a leading role on device performance and stability.^45,52,53^

A systematic mixing of HTMs, consisting of spiro-OMeTAD and other hole-transporting materials, is considered a potential solution to address the limitations of spiro-OMeTAD. These composite HTM materials provide several advantages, including enhanced oxidation of spiro-OMeTAD, improved doping efficiency, increased molecular packing within the spiro-OMeTAD-based hole-transporting layer, regulating light absorption, resistance to moisture penetration, and surface defect passivation. These benefits collectively contribute to improved performances and operational stability of PSCs.^54–56^ For examples, Liu et al. demonstrated a composite HTM comprising spiro-OMeTAD and acceptor–donor–acceptor (A–D–A) type molecules, which demonstrated superior passivation effects and high hole mobility, resulting in enhanced efficiency as well as stability in contrast to the single component HTM-based devices.^46^ Similarly, Park and colleagues presented that incorporating 5 mol % of donor-π spacer-donor (D-π-D) type small molecules into spiro-OMeTAD modified the intermolecular interaction and facilitated efficient hole extraction to increase the device efficiency and stability, where 90% PCE was sustained during 1200 h of functioning without encapsulation.^57^ Recently, Chang et al. reported blended hole-transporting materials incorporating spiro-OMeTAD and electron-deficient benzo­[*g*]­quinoxaline-conjugated small molecules to enhance the PSC effectiveness, film morphology, and charge transport.

Although composite HTMs consisting of spiro-OMeTAD with HTMs show promise in addressing the shortcoming properties of spiro-OMeTAD, developing appropriate composite materials that fulfill the stringent requirements of PSCs remains challenging. In this study, we designed two structurally simple and cost-effective conjugated isomeric small molecules, 2,7-OPOT and 3,6-OPOT, based on a 9,10-dimethoxyphenanthrene core within a D-π-D framework. The two molecules, 2,7-OPOT and 3,6-OPOT, are position isomers with the same molecular formula and functional groups but substituents at different positions of the main carbon skeleton. The prefixes 2,7- and 3,6- represent the positions of the donor moieties attached to the 9,10-dimethoxyphenanthrene core. The molecular structures of 2,7-OPOT, 3,6-OPOT, and spiro-OMeTAD are illustrated in Figure 1. The 9,10-dimethoxyphenanthrene core units are easy to synthesize and scale up with excellent purity from inexpensive starting materials. More interestingly, even though this core unit is a small organic molecule, this core enables the design of new materials incorporating multiple defect-passivating functionalities, such as methoxy and amino group. Another key advantage of this core structure lies in its versatility for structural engineering, allowing for the introduction of identical donor units at different positions within the framework. This provides an effective strategy to fine-tune the highest occupied molecular orbital (HOMO) energy level of the material for optimal alignment with the valence band edge of perovskite by combining suitable donor units. To the best of our knowledge, this is the first report of the utilization of 9,10-dimethoxyphenanthrene core-based molecules in composite HTMs for PSCs. The OPOT design has the advantage of multiple defect passivating functionalities such as methoxy and amino groups. These OPOTs were mixed with spiro-OMeTAD, hereafter named the composites S-2,7-OPOT and S-3,6-OPOT, respectively, to function as composite HTMs. The OPOTs exhibit superior solubility in organic solvents and good miscibility with spiro-OMeTAD solution, which is crucial for producing high-quality device films. Composite HTMs were prepared by mixing a dopant-free OPOT solution with a spiro-OMeTAD solution. The photovoltaic parameters of these composite HTMs were thoroughly investigated across various mixing ratios. The optimized mixing molar ratio for achieving the best performance was determined to be 0.18 (OPOTs/spiro-OMeTAD). The composite HTMs have shown several advantageous properties, including appropriate energy levels, superior thermal stability, resistance to moisture penetration, enhanced hole mobility, passivating defects, and effective hole extraction. The champion device with an S-3,6-OPOT-based composite HTM achieved a high PCE of 18.8%, outperforming both S-2,7-OPOT (18.6%) and standard spiro-OMeTAD (17.7%). Furthermore, the S-3,6-OPOT-based PSC device maintained above 81% of its initial PCE following a storage of 60 days under ambient condition (relative humidity, RH ca. 45%), while the spiro-OMeTAD-based PSC device plunged to ∼70%. This work emphasizes the potential of composite HTM based on spiro-OMeTAD and judiciously designed cost-effective hole-selective small organic molecules to improve the efficiency of the device. By reduction of the dopant loading of spiro-OMeTAD, these composites represent a promising model for future designs aimed at achieving high PCE and improved stability.

**Figure 1:**
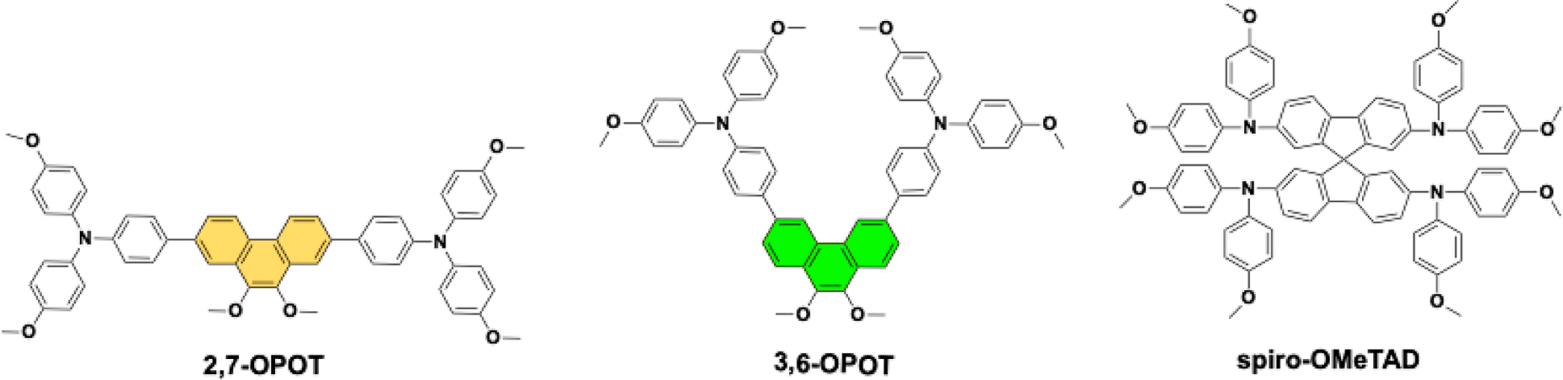
Molecular structures of 2,7-OPOT, 3,6-OPOT, and spiro-OMeTAD.

## Results and Discussion

The isomeric pair of 2,7-OPOT and 3,6-OPOT was synthesized (Scheme 1) in good yields via the Suzuki coupling reaction between the donor, 4-methoxy-*N*-(4-methoxyphenyl)-*N*-(4-(4,4,5,5-tetramethyl-1,3,2-dioxaborolan-2-yl)­phenyl)­aniline (M7) and core compounds of 2,7-dibromo-9,10-dimethoxyphenanthrene (M3) and 3,6-dibromo-9,10-dimethoxyphenanthrene (M9), respectively. The comprehensive synthetic pathways are presented in Scheme S1. The purity and identity of OPOTs were thoroughly analyzed by ^1^H and ^13^C NMR spectroscopies (Figures S1–S4), high-resolution mass spectrometry, and single crystal analysis (Figures S5–S7 and Table S1). The material costs for both OPOTs were calculated and are tabulated in Tables S2 and S3.

**Scheme 1:**
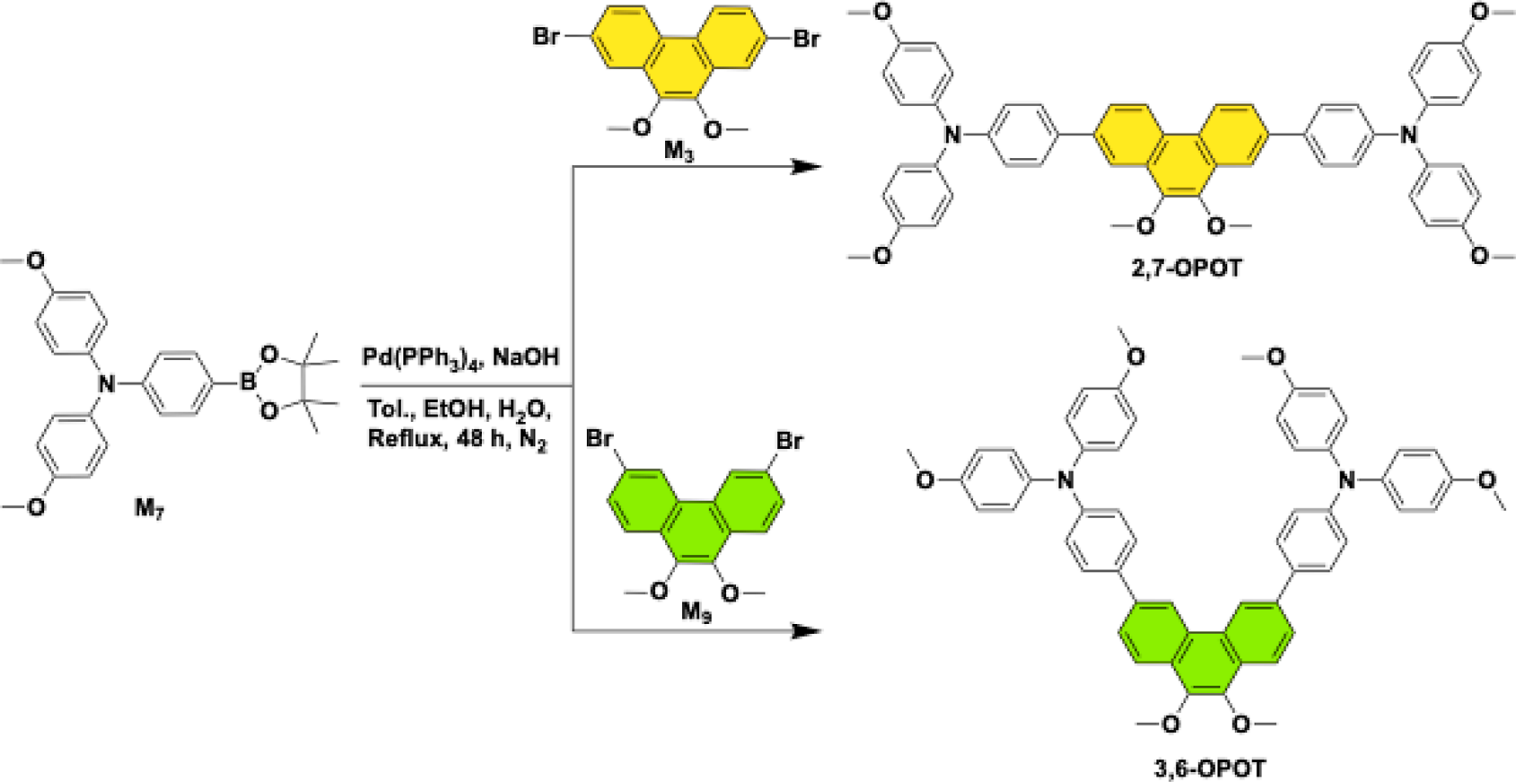
Synthetic Routes of 2,7-OPOT and 3,6-OPOT

The UV–vis absorption spectra were measured for the films of composite HTMs as well as spiro-OMeTAD with an optimized molar ratio of 0.18 (OPOTs/spiro-OMeTAD) on a quartz substrate (Figure 2a). The composite HTMs exhibited two prominent absorption bands: 305 and 376 nm for S-2,7-OPOT, and 307 and 374 nm for S-3,6-OPOT, corresponding to π–π* transitions.^60^ These absorption spectra closely resemble those of spiro-OMeTAD, which shows absorption maxima at 308 and 377 nm. The optical band gap (*E*_g_) was estimated using the Tauc plots (Figure 2b). S-2,7-OPOT displays a slightly smaller band gap compared to S-3,6-OPOT and spiro-OMeTAD. The lowest unoccupied molecular orbital (LUMO) levels of the HTMs were calculated using the equation *E*_LUMO_ = *E*_HOMO_ + *E*_g_.^61^ The LUMO levels were found to be −1.98 eV, −1.97 eV, and −1.96 eV for S-2,7-OPOT, S-3,6-OPOT, and spiro-OMeTAD, respectively. These LUMO levels are sufficiently high enough to block electron flow from the perovskite to the HTMs, thereby preventing charge recombination.^62,63^ The optical data are collected in Table 1. A representative device structure of n-i-p type PSC and the energy level diagram constituting different layers of the device are given in Figure 3.

**Figure 2:**
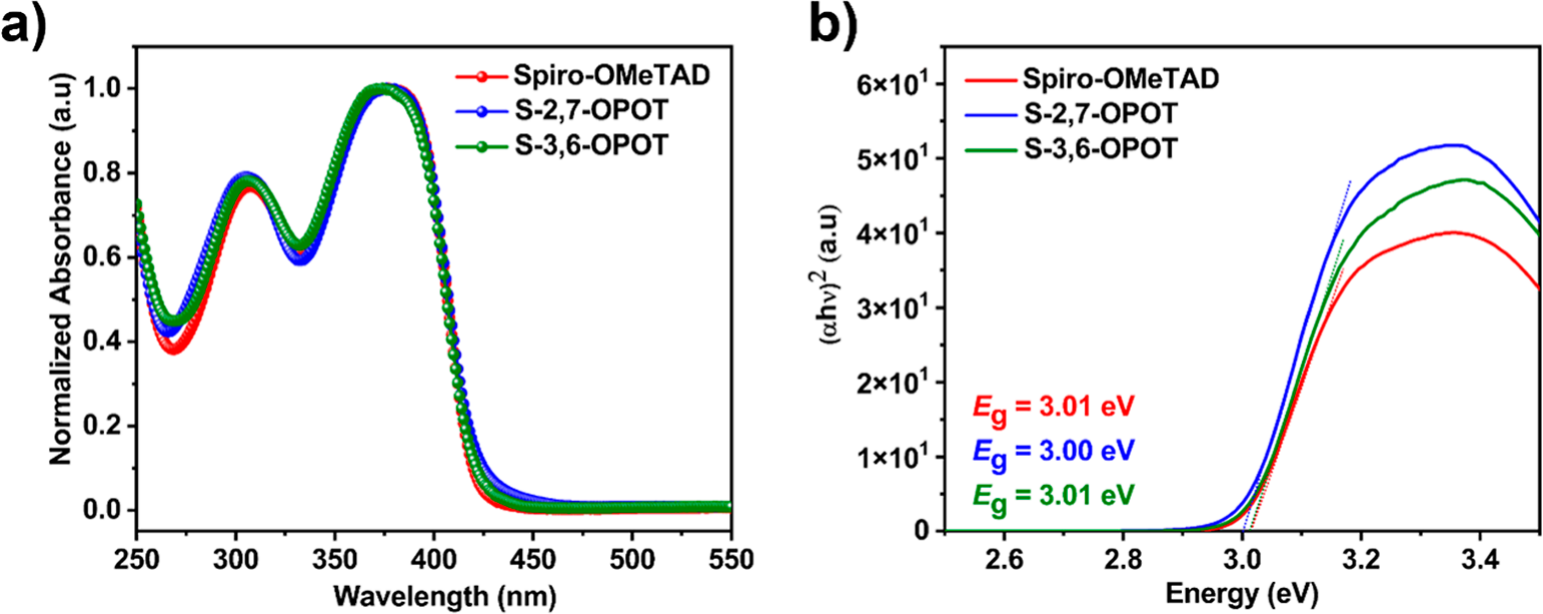
(a) UV–vis absorption spectra of spiro-OMeTAD, S-2,7-OPOT, and S-3,6-OPOT films. (b) Tauc plots of spiro-OMeTAD, S-2,7-OPOT, and S-3,6-OPOT.

**Table 1:** Optical Properties, Energy Levels, and Hole Mobility Measurements by SCLC Methods of Composite HTMs and Spiro-OMeTAD

HTM	*E*_HOMO_^a^ [eV]	λ_max, abs_ ^b^ [nm]	*E*_g_^c^ [eV]	*E*_LUMO_^d^ [eV]	μ^e^ [cm^2^ V^–1^ s^–1^]	*V*_TFL_^f^ [V]	*N*_t_^g^ [cm^–3^]
S-2,7-OPOT	–4.98	305, 376	3.00	–1.98	3.81 × 10^–4^	0.708	4.14 × 10^15^
S-3,6-OPOT	–4.98	307, 374	3.01	–1.97	5.55 × 10^–4^	0.664	3.88 × 10^15^
spiro-OMeTAD	–4.97	308, 377	3.01	–1.96	1.96 × 10^–4^	0.745	4.36 × 10^15^

a Determined from the photoemission spectroscopy measurements of HTM films.

b Measured from the UV–vis spectroscopy of HTM films.

c Estimated by Tauc plots of the absorption spectra in solid films.

d Calculated by the equation *E*_LUMO_ = *E*_HOMO_ + *E*_g_.

e Hole mobility.

f Trap-filled-limit voltages.

g Defect state density of HTMs.

**Figure 3:**
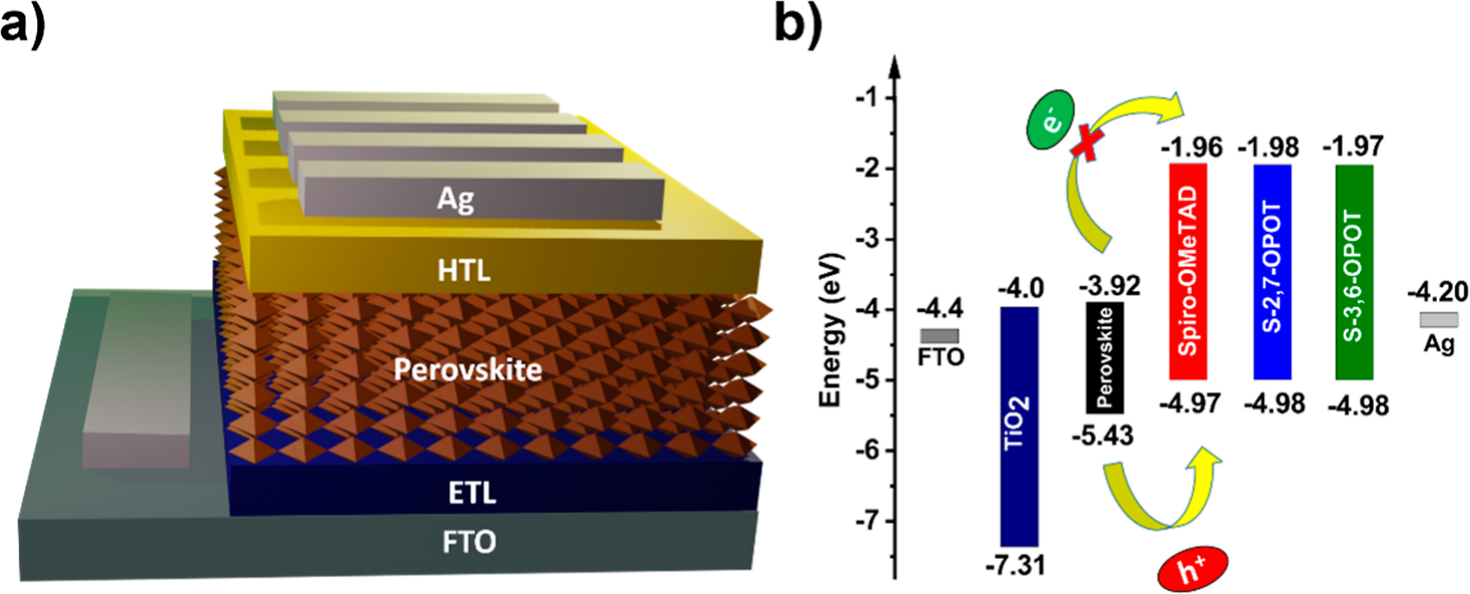
(a) Representative device structure of the PSC. (b) Energy level diagram of different layers of n-i-p PSCs.

The HOMO levels of composite HTMs and spiro-OMeTAD were estimated from the photoemission spectroscopy (PES) measurement of the films prepared on an indium tin oxide substrate. The HOMO levels of S-2,7-OPOT and S-3,6-OPOT are the same (−4.98 eV), slightly deeper than those of spiro-OMeTAD (−4.97 eV), as shown in Figure S8. These values indicate that the composite HTMs possess suitable HOMO levels for effective hole extraction from the perovskite, which has a valence band edge value of −5.43 eV.^13^ Furthermore, the individual HOMO levels of 2,7-OPOT and 3,6-OPOT were measured at −5.12 eV and −5.19 eV, respectively, with the corresponding PES spectra provided in Figure S9.

The hole mobilities (μ), trap-filled-limit voltages (*V*_TFL_), and defect state density (*N*_t_) of S-2,7-OPOT, S-3,6-OPOT, and spiro-OMeTAD were determined using space charge limited current (SCLC) measurements (Figure S10), in regard to hole-only devices with a configuration of the FTO/PEDOT:PSS/MAPbI_3_/HTM/Ag electrode. The hole mobilities of S-2,7-OPOT and S-3,6-OPOT were found to be 3.81 × 10^–4^ and 5.55 × 10^–4^ cm^2^ V^–1^ s^–1^, respectively, which are higher than that of spiro-OMeTAD (1.96 × 10^–4^ cm^2^ V^–1^ s^–1^). The higher hole mobilities of the composite HTMs reveal that mixing small molecules (2,7-OPOT and 3,6-OPOT) with spiro-OMeTAD enhances the hole extraction process and effectively suppresses nonradiative recombination at the perovskite/HTM interfaces. The *V*_TFL_ at the perovskite/HTM interfaces was also significantly reduced with the composite HTMs of S-2,7-OPOT (0.708 V) and S-3,6-OPOT (0.664 V) compared to that of spiro-OMeTAD (0.745 V). Furthermore, the lower *N*_t_ values of S-2,7-OPOT (4.14 × 10^15^ cm^–3^) and S-3,6-OPOT (3.88 × 10^15^ cm^–3^) as compared to a single component of spiro-OMeTAD (4.36 × 10^15^ cm^–3^) made it evident that the composite HTM, especially S-3,6-OPOT, well suppressed the deep level defects of trap-assisted charge recombination losses, thereby improving charge transport properties.^64^

The thermal properties of HTMs were assessed using thermogravimetric analysis (TGA) and differential scanning calorimetry (DSC), as illustrated in Figures S11 and S12. Generally, the change in slope or peaks in the TGA curves indicates the occurrence of weight loss of the material during the thermal events such as decomposition, volatilization, and phase transition. These materials exhibited excellent thermal stability, with decomposition temperatures exceeding 400 °C, as summarized in Table S4. 2,7-OPOT and 3,6-OPOT showed a decomposition temperature (*T*_d_) of 417 and 403 °C, respectively. The thermal stability was further improved for their corresponding composite HTMs, with *T*_d_ values of 441 °C for S-2,7-OPOT and 438 °C for S-3,6-OPOT, comparable to the *T*_d_ of 441 °C recorded for the single component spiro-OMeTAD. The degradation pattern of composite HTMs is more similar to the spiro-OMeTAD than OPOTs since the major component in the composite HTM is spiro-OMeTAD. The *T*_g_ of the composites S-2,7-OPOT and S-3,6-OPOT were measured at 120 °C from the DSC. A *T*_g_ value of 125 °C was achieved for the spiro-OMeTAD, which is in agreement with the previous report.^65^ These findings revealed that the composite HTMs possess good thermal stability, making them appropriate for use in PSCs. Powder X-ray diffraction measurements were conducted for the thin films of S-2,7-OPOT, S-3,6-OPOT, and spiro-OMeTAD. The composite HTMs showed broad diffraction patterns similar to those of spiro-OMeTAD, confirming the amorphous nature of the composite HTMs (Figure S13).

The hole extraction properties at the perovskite/HTM interface were studied by employing steady-state photoluminescence (PL) and time-resolved photoluminescence (TRPL) measurements (Figure 4a,b). The PL intensity of the perovskite is more effectively quenched by the composite HTMs compared to the single component spiro-OMeTAD, indicating superior hole extraction efficiency.^66^ TRPL data revealed significantly shorter average decay times (τ_avg_) for the bilayers of perovskite/S-3,6-OPOT (25.45 ns), perovskite/S-2,7-OPOT (29.12 ns), and perovskite/spiro-OMeTAD (36.32 ns). In contrast, the pristine perovskite layer shows a much longer decay time (85.64 ns). These results further confirm effective charge carrier transfer at the perovskite/HTM interface.

**Figure 4:**
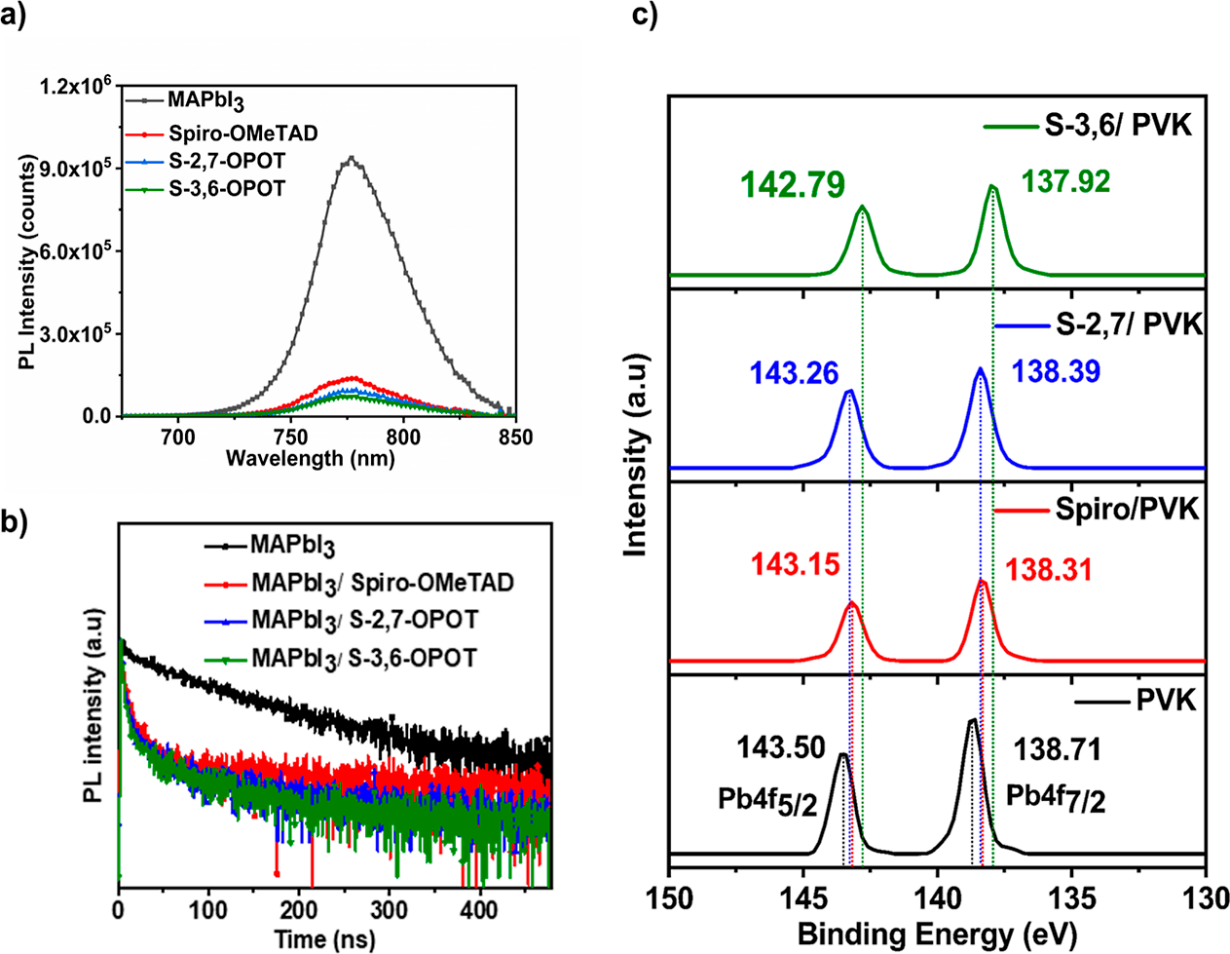
(a) Steady-state PL, (b) TRPL decay curves, and (c) XPS Pb 4f signals of perovskite films with and without HTMs.

X-ray photoelectron spectroscopy (XPS) was performed to explore the interactions between the composite HTMs and the perovskite. The uncoordinated Pb^2+^ and interstitial iodide vacancies are known to be the major sources of deep-level defects that are detrimental to the performance and durability of PSCs.^67^ Thus, the binding energy changes of Pb 4f and I 3d signals were examined in the FTO/MAPbI_3_/HTMs device structure to assess the passivation effect of the composite HTMs on the perovskite. For pristine perovskite, the binding energies corresponding to Pb 4f_5/2_ and Pb 4f_7/2_ are 143.50 and 138.71 eV, respectively, consistent with literature values.^68^ Upon coating with spiro-OMeTAD, S-2,7-OPOT, and S-3,6-OPOT on perovskite, the Pb 4f binding energies shifted to lower values (Figure 4c), with the shifts being most pronounced for S-3,6-OPOT. A similar trend of binding energy shift was observed for the I 3d signals (Figure S14). These energy shifts are attributed to the Lewis acid–base interactions of the electron-rich nature of S-3,6-OPOT and S-2,7-OPOT with uncoordinated species at the interfaces and boundaries of the perovskite.^69,70^ Composite HTMs (S-2,7-OPOT, S-3,6-OPOT) and standard spiro-OMeTAD consist of the same passivating groups such as methoxy and amino groups, but the strength and extent of defect passivating interactions with the perovskite layer might be different according to their configuration and structural features.^71–73^ In this case, composite HTM S-3,6-OPOT exhibits stronger defect passivating interactions with Pb^2+^ than the other two HTMs as indicated by the XPS results.

The efficiency of hole-transport layer materials also relies on the quality and uniformity of the film.^74^ The film morphology and quality were analyzed by using scanning electron microscopy (SEM). Top-view SEM images of the perovskite layer with and without HTMs are shown in Figure 5a–d with a device configuration of FTO/perovskite/HTM. The composite HTMs demonstrated uniform coverage over the perovskite layer, with no significant morphological differences compared to the single-component HTM. Furthermore, the hydrophobic nature of HTMs was examined by water contact angle measurements. The contact angles for spiro-OMeTAD, S-2,7-OPOT, and S-3,6-OPOT were measured to be 78.4°, 89.8°, and 90.5°, respectively. The larger contact angles observed for the composite HTMs suggest superior protection of the perovskite layer against moisture.^75^ This improvement can be linked to the smoother surface and increased hydrophobicity of the composite films compared to that based on the spiro-OMeTAD based film (Figure 5e–g).

**Figure 5:**
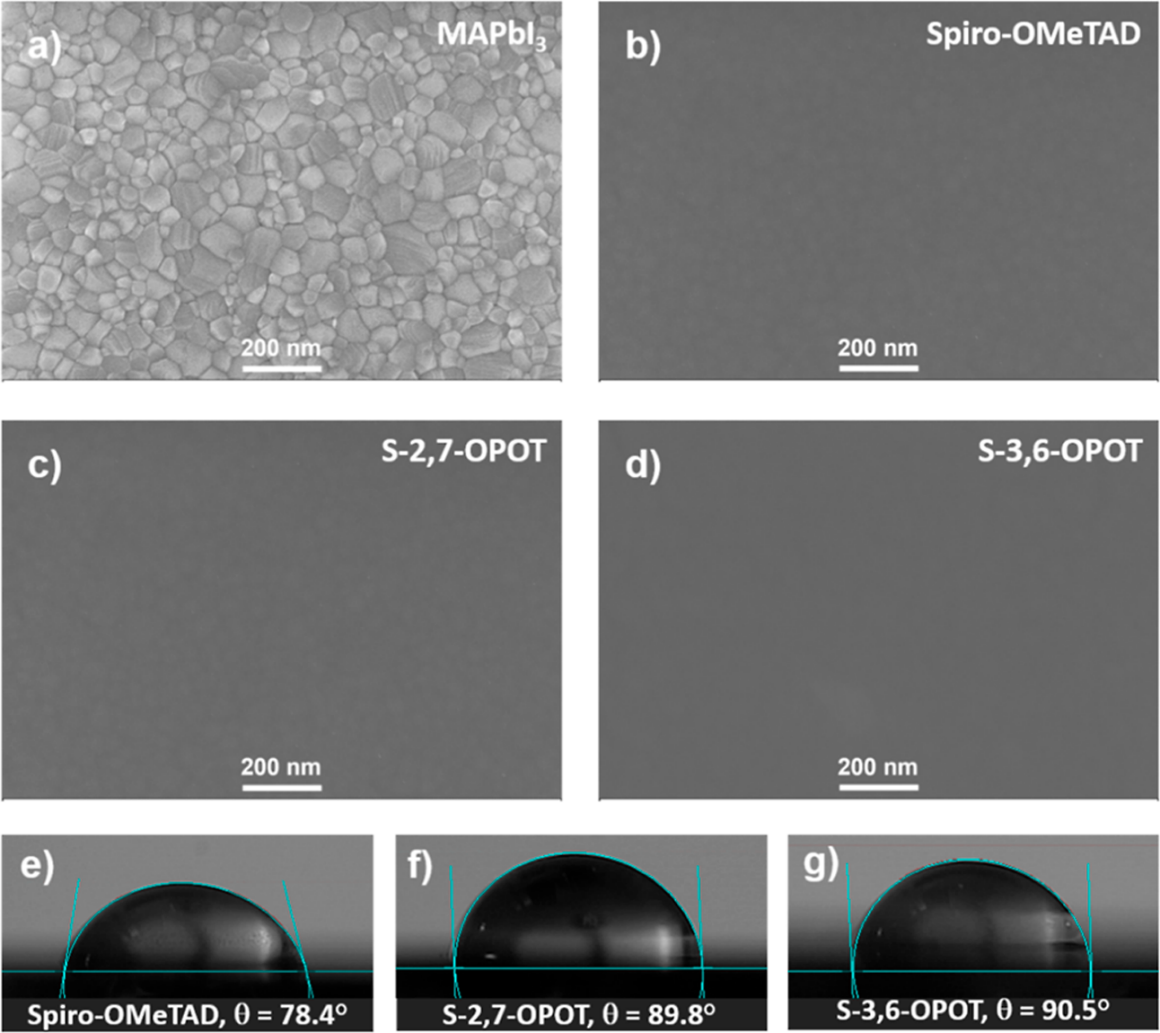
(a–d) Top view SEM images of perovskite and HTMs spiro-OMeTAD, S-2,7-OPOT, and S-3,6-OPOT on top of perovskite. (e–g) Water contact angles of perovskite/HTM bilayer samples.

The photovoltaic performance of n-i-p type PSCs with the device configuration of FTO/cp-TiO_2_/mp-TiO_2_/CH_3_NH_3_PbI_3_ (MAPbI_3_)/HTM/Ag was investigated with composite HTMs of S-2,7-OPOT and S-3,6-OPOT alongside spiro-OMeTAD as a control device. Detailed studies on the device performance of composite HTMs with different ratios of spiro-OMeTAD/OPOTs were performed. The optimized molar ratio of OPOT/spiro-OMeTAD was found to be 0.18. The current density–voltage (*J*–*V*) curves under standard AM 1.5G (100 mW cm^–2^) illumination of the champion devices are shown in Figure 6a, and the corresponding photovoltaic parameters are collected in Table 2. The PSCs constituting S-3,6-OPOT achieved a high PCE of 18.8% (*V*_oc_ = 1.05 V, *J*_sc_ = 23.9 mA cm^–2^ and FF = 74.9%), while devices with S-2,7-OPOT achieved a PCE of 18.6% (*V*_oc_ = 1.05 V, *J*_sc_ = 23.6 mA cm^–2^ and FF = 75.1%). Both devices based on composite HTMs reveal significant performance enhancements compared to the control device with spiro-OMeTAD, which achieved a PCE of 17.7% (*V*_oc_ = 1.03 V, *J*_sc_ = 23.2 mA cm^–2^ and FF = 74.8%). The incident photon-to-current conversion efficiency (IPCE) spectra and the corresponding integrated *J*_sc_ are shown in Figure 6b. The devices displayed good photo responses, as indicated by the plateau across the 400–800 nm range. The integrated *J*_sc_ values derived from the IPCE curves align closely with the trends observed in the *J*–*V* measurements. Dark current measurements (Figure S15) were also conducted to evaluate the charge transport properties and their influence on device performance. The composite HTM-based devices exhibited lower leakage currents at 0 V of applied bias, suggesting reduced defect density and improved charge transport.^58^ These results indicate lower leakage current, reduced defect density, improved charge transport, and in effect more charge carriers are available to enhance the voltage. The series resistance (*R*_s_) and shunt resistance (*R*_sh_) were extracted from the inverse slope near the open circuit (*V*_oc_) and short circuit current (*I*_sc_), respectively, of the corresponding *I*–*V* curves. As shown in Table S5, the composite HTM materials displayed lower *R*_s_ and higher *R*_sh_ compared to the corresponding spiro-OMeTAD HTM-based PSCs, affirming that the charge recombination at the interfaces or boundaries and the defects or imperfections that are responsible for trap-assisted leakage in the devices are well passivated using composite HTM materials. These results are well conceded with dark current data, indicating a low leakage current at applied bias of 0 V from the composite HTM materials-based PSCs.

**Figure 6:**
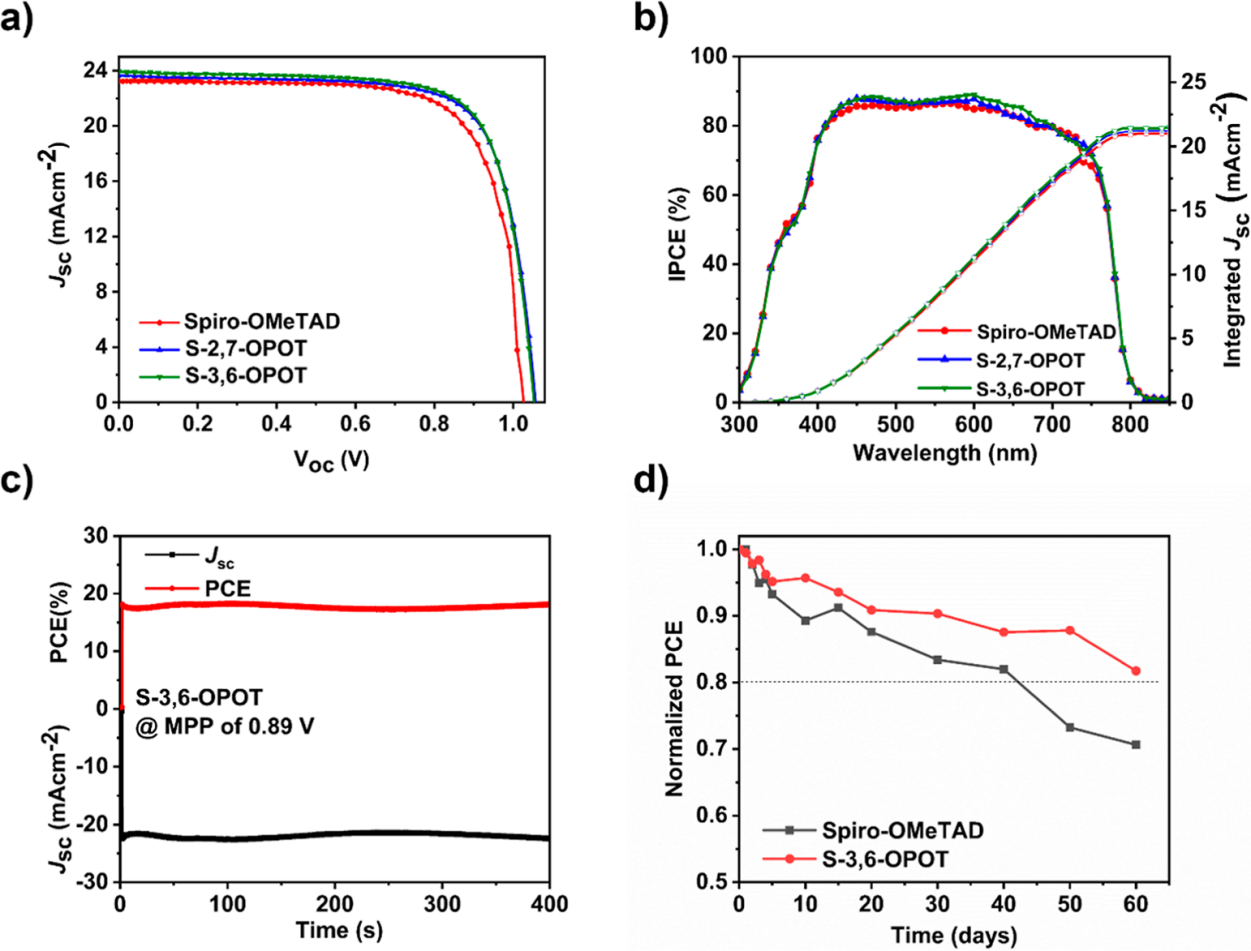
(a) *J*–*V* curves of PSCs under the standard AM 1.5G (100 mW cm^–2^) illumination. (b) The corresponding IPCE curves. (c) The stabilized power efficiency output and photocurrent density measurements at the maximum power point condition. (d) Long-term stability plots of PSCs.

**Table 2:** Photovoltaic Parameters of PSCs Based on Spiro-OMeTAD, S-2,7-OPOT, and S-3,6-OPOT HTMs^a^

HTM	*V*_oc_ [V]	*J*_sc_ [mA cm^–2^]	FF [%]	PCE [%]
spiro-OMeTAD	1.02 ± 0.02 (1.03)	23.36 ± 0.37 (23.2)	71.64 ± 0.03 (74.8)	17.10 ± 0.50 (17.7)
S-2,7-OPOT	1.04 ± 0.01 (1.05)	23.57 ± 0.62 (23.6)	73.40 ± 0.03 (75.06)	17.93 ± 0.46 (18.6)
S-3,6-OPOT	1.04 ± 0.02 (1.05)	23.68 ± 0.57 (23.9)	74.13 ± 0.02 (74.92)	18.16 ± 0.39 (18.8)

a The average was based on 10 devices, and the data of champion devices are shown in parentheses.

In this study, the composite HTM materials exhibited higher hole mobilities, lower trap-filled-limit voltages, and reduced defect state density compared with the corresponding single component spiro-OMeTAD HTM. These findings are further supported by PL and TRPL measurements, which confirm more effective charge carrier transfer at the perovskite/HTM interface. We attribute these improvements to the OPOT-based composite HTMs, which enhance intermolecular interactions within the spiro-OMeTAD-based hole transport layer and provide effective surface passivation. This not only facilitates efficient hole extraction but also suppresses nonradiative recombination at the perovskite/HTM interfaces and mitigates deep-level defects associated with trap-assisted charge recombination losses. Furthermore, the *R*_s_ and *R*_sh_ values are well conceded with the dark current characteristics, indicating a low leakage current at 0 V bias in devices employing the composite HTMs. Notably, devices based on S-3,6-OPOT demonstrated superior performance compared to those with S-2,7-OPOT, which could be attributed to the structural differences influencing molecular packing and interfacial interactions within the composite HTMs. Consequently, these favorable enhancements collectively lead to improved photocurrents, photovoltages, and overall higher PCEs in the composite HTM-based PSCs.

The stabilized power efficiency output and current density for the device based on S-3,6-OPOT were measured at the maximum power point, as shown in Figure 6c. It is evident that the stabilized power output and current density remained constant over a period of 400 s even under high humid environment of relative humidity (RH) > 80%, indicating the stable performance of the device. The long-term stability was investigated by exposing the devices with HTMs S-3,6-OPOT and spiro-OMeTAD to 45% RH without encapsulation in air. The PSCs with S-3,6-OPOT retained ∼81% of its initial efficiency even after 60 days, whereas the spiro-OMeTAD based PSC dropped to ∼70% of its initial PCE following the same duration (Figure 6d). These results confirm the improvement in durability of devices with the employment of composite HTM S-3,6-OPOT. Moisture and oxygen are known to adversely affect the stability of perovskite materials, leading to their degradation and diminished device performance. Incorporating HTMs with optimal hydrophobicity atop the perovskite layer can serve as an effective barrier against moisture penetration, thereby enhancing the stability of PSCs. In the current study, the larger water contact angle observed for the S-3,6-OPOT/perovskite surface indicates improved hydrophobic protection, which likely mitigates moisture-induced degradation of the perovskite layer and contributes to the enhanced operational stability of the corresponding PSCs.

The reproducibility of the performance was evaluated by fabricating ten individual PSCs for each HTM: spiro-OMeTAD, S-2,7-OPOT, and S-3,6-OPOT. As shown in Figure S16, the statistical distributions of *V*_oc_, *J*_sc_, FF, and PCE signify the reliability of the data for PSCs. As aforementioned, the superior photovoltaic performance of PSCs based on the composite HTMs compared to the counterpart of spiro-OMeTAD can be ascribed to a synergistic effect arising from effective intermolecular interactions and passivation, leading to smoother surfaces with lower defect densities, favorable hole extraction with enhanced hole mobility, and suppression of the nonradiative charge recombination. PSCs hold great potential to substitute for the existing commercial photovoltaic technologies, provided that key challenges related to stability, scalability, and cost are well addressed. Therefore, this study employs lower dopant loading of spiro-OMeTAD by designing composite HTMs, offering a more cost-effective and stable alternative. This approach is particularly relevant for advancing industrial applications and large-scale production of PSCs.

## Conclusions

In conclusion, we successfully synthesized two structurally simple and material-cost-effective conjugated isomeric small organic molecules, 2,7-OPOT and 3,6-OPOT, with a 9,10-dimethoxyphenanthrene core in a D-π-D structure. These new small molecules were mixed with spiro-OMeTAD to produce composite HTMs of S-2,7-OPOT and S-3,6-OPOT, with the aim to improve the perovskite surface passivation and enhance hole extraction and hole mobility and against moisture penetration, which lead to the performance enhancement and operational stability of PSCs. The solutions of OPOTs based HTMs have shown good miscibility with spiro-OMeTAD. Notably, the composite HTMs demonstrated a notable enhancement in the optoelectronic, thermal, and morphological properties compared to the counterpart of single component spiro-OMeTAD. The devices based on S-3,6-OPOT composite HTM achieved a PCE of 18.8% with *J*_sc_ of 23.9 mA cm^–2^, *V*_oc_ of 1.05 V and FF of 74.92%, which surpasses the PCEs of S-2,7-OPOT (18.6%) and standard spiro-OMeTAD (17.7%)-based devices. The enhanced photovoltaic performance of PSCs incorporating composite HTMs, in contrast to those using spiro-OMeTAD, can be attributed to a synergistic effect stemming from effective intermolecular interactions and passivation, which result in smoother surface morphology, reduced defect densities, improved hole extraction, increased hole mobility, and a reduction in nonradiative charge recombination. The long-term stability for the PSCs fabricated with S-3,6-OPOT retained more than 81% of its initial performance after 60 days of exposure to 45% RH without encapsulation in air, which is considerably better compared to the control device with HTM-spiro-OMeTAD. These findings confirm that a composite HTM mixing of organic small molecule with spiro-OMeTAD is a prospective strategy to enhance the photovoltaic parameters and operational stability of PSCs with lower dopant loading amount and expenses of spiro-OMeTAD.

## Experimental Section

### Materials and Synthesis

Scheme 1 outlines the synthetic procedures for the preparation of two new OPOT molecules. Compounds M_3_,^76^ M_7_,^77^ and M_9_ were synthesized according to the previous literature procedures.^78^

### Synthesis of 2,7-OPOT

In an oven-dried Schlenk flask, M_3_ (0.300 g, 0.757 mmol), M_7_ (0.977 g, 2.27 mmol), Pd­(PPh_3_)_4_ (0.175 g, 0.151 mmol), and NaOH (0.182 g, 4.54 mmol) were weighed and degassed three times. A mixture of solvents containing toluene, ethanol, and deionized water with 12, 8, and 4 mL (3:2:1, v/v), respectively, purged earlier by N_2_, was transferred to the Schlenk flask. The solution was allowed to reflux for 48 h under a N_2_ atmosphere. After completion of the reaction, solvent was evaporated, and the residual was extracted with 100 mL of ethyl acetate. The crude was subjected to silica column chromatographic separation using 15% ethyl acetate/hexane eluent to obtain a pale greenish-yellow solid in 79% yield. ^1^H NMR (400 MHz, DMSO-*d*_6_): δ (ppm): 8.79 (d, *J* = 8.8 Hz, 2H), 8.28 (d, *J* = 1.6 Hz, 2H), 7.88 (dd, *J* = 8.8 Hz, and *J* = 2 Hz, 2H), 7.69 (d, *J* = 8.8 Hz, 4H), 7.09 (d, *J* = 8.8 Hz, 8H), 6.95 (d, *J* = 8.8 Hz, 8H), 6.91­(d, *J* = 8.4 Hz, 4H), 4.05 (s, 6H), 3.76 (s, 12H). ^13^C NMR (100 MHz, CDCl_3_): δ (ppm) 156.09, 148.48, 144.54, 141.03, 139.11, 132.97, 129.42, 127.96, 126, 80, 124.83, 123.30, 120.94, 119.32, 114.90, 61.14, 55.66. MALDI-HRMS: (M^+^) calcd for C_56_H_48_N_2_O_6_, 844.3512; found, 844.3536.

### Synthesis of 3,6-OPOT

Compound 3,6-OPOT was synthesized by following a procedure similar to that for 2,7-OPOT using M_9_ (0.400 g, 1.01 mmol), M_7_ (1.31 g, 3.03 mmol), Pd­(PPh_3_)_4_ (0.231 g, 0.200 mmol), and NaOH (0.240 g, 0.600 mmol). After silica column chromatographic purification using 15% ethyl acetate/hexane, a pale-yellow solid (75%) was obtained. ^1^H NMR (400 MHz, DMSO-*d*_6_): δ (ppm): 9.04 (s, 2H), 8.18 (d, *J* = 8.8 Hz, 2H), 7.93­(dd, *J* = 8.8 Hz, *J* = 1.2 Hz, 2H), 7.80 (d, *J* = 8.8 Hz, 4H), 7.08 (d, *J* = 8.8 Hz, 8H), 6.95 (d, *J* = 8.8 Hz, 8H), 6.91­(d, *J* = 8.8 Hz, 4H), 4.04­(s, 6H), 3.76 (s, 12H). ^13^C NMR (100 MHz, CDCl_3_): δ (ppm) 156.08, 148.44, 143.89, 141.04, 138.47, 133.37, 129.17, 128.09, 126.78, 126.02, 122.80, 121.03, 120.38, 114.90, 61.18, 55.66. MALDI-HRMS: (M^+^) calcd for C_56_H_48_N_2_O_6_, 844.3512; found, 844.3515.

### Device Fabrication and Performance Measurement

PSCs were fabricated following a previously reported procedure.^13^ Detailed procedures are described in the Supporting Information. Briefly, electron transport layers of TiO_2_ were deposited on FTO substrates, as described in the literature procedure. These FTO/TiO_2_ substrates were then transferred into a glovebox for the deposition of the active material. A 1.4 M solution of MAPbI_3_ was prepared in DMF/DMSO mixture (9:1, v/v ratio) and then spin-coated via a one-step deposition technique at 4000 rpm for 25 s. A total of 150 μL of anhydrous chlorobenzene was rapidly dripped on top of the substrate at the 10th second. The perovskite-spin-coated substrates were then annealed at 100 °C for 5 min. Subsequently, a hole-transporting material was deposited at 4000 rpm for 30 s. The composite HTM solution of S-2,7-OPOT and S-3,6-OPOT was prepared by mixing their individual solutions with a spiro-OMeTAD solution in a 3:1 v/v ratio. A spiro-OMeTAD solution with dopant was used as a control. The PSC device fabrication with a structure of FTO/TiO_2_/CH_3_NH_3_PbI_3_/HTM/Ag was completed with thermal evaporation of ∼80 nm thick silver onto the HTM layer at a pressure of 5 × 10^–7^ Torr using a metal mask with an active area of 0.04 cm^2^. The solar cell efficiencies were measured with simulated AM 1.5G irradiation of 100 mW cm^–2^ (1 sun). The *J*–*V* measurements were carried out with a 0.04 cm^2^ active area mask.

## Supplementary Material





## Notes

Comprehensive experimental methods and characterization for the new molecules, crystal structure analysis, device fabrication procedures, and additional experimental data for physical characterization of materials and devices (PDF)
(CIF)
