# Supplementary material for: Composite Hole-Transporting Materials Based on 9,10-Dimethoxyphenanthrene Cores and Spiro-OMeTAD for Efficient and Stable Perovskite Solar Cells
Source: ACS Omega. 2025 May 14;10(20):20638–48. doi: 10.1021/acsomega.5c01513 (PMC12120597; doi:10.1021/acsomega.5c01513)
Supplement: Supplementary file 1 [file ao5c01513_si_001.pdf]

Supporting Information

Composite Hole-Transporting Materials Based on 9,10-Dimethoxyphenanthrene Cores and Spiro-OMeTAD for Efficient and Stable Perovskite Solar Cells

Jijitha Vailassery,[a,b,c‡] Gebremariam Zebene Wubie,[a‡] Jia-Wei She,[a] Wen-Ti Wu,[a] Hsiao-hua Yu,[a] and Shih-Sheng Sun[*a]

[a]Institute of Chemistry, Academia Sinica, No.128, Academia Road, Sec. 2, Nankang, Taipei 115, Taiwan, R.O.C.

[b]Taiwan International Graduate Program, Sustainable Chemical Science and Technology.

[c]Department of Applied Chemistry, National Yang Ming Chiao Tung University, Hsinchu, Taiwan 300, R.O.C.

Corresponding Author

Shih-Sheng Sun, Email: sssun@chem.sinica.edu.tw

‡These authors contributed equally.

Table of Contents

Materials and Characterization Methods……………………………………………………….4-5

Synthetic Schemes and Experimental Procedures……………………………………….......6

Scheme S1. Synthesis of 2,7-OPOT and 3,6-OPOT………………………………………...6

Synthesis of 4,4'-(9,10-dimethoxyphenanthrene-2,7-diyl)bis(*N*,*N*-bis(4-methoxyphenyl)aniline) (2,7-OPOT)………………………….…………………………....7

Synthesis of 4,4'-(9,10-dimethoxyphenanthrene-3,6-diyl)bis(*N*,*N*-bis(4-methoxyphenyl)aniline) (3,6-OPOT)…………………………………………...……..........7

Figure S1. ^{1}H NMR of 2,7-OPOT (400 MHz, DMSO-d6) ………………………….........8

Figure S2. ^{13}C NMR of 2,7-OPOT (100 MHz, $CDCl_3$) ……………………………...…...8

Figure S3. ^{1}H NMR of 3,6-OPOT (400 MHz, DMSO-d6) ……………………………….....9

Figure S4. ^{13}C NMR of 3,6-OPOT (100 MHz, $CDCl_3$) ……………………...………....9

Figure S5. Single crystal structure of 3,6-OPOT…………………………………...……...10

Figure S6. Molecular packing of 3,6-OPOT in a unit cell………………………...………10

Figure S7. CH-π interactions extracted from single crystal analysis of 3,6-OPOT……….......11

Table S1. Crystal data and structure refinement for 3,6-OPOT…………………...…....11-12

Table S2. Material cost for the synthesis of 1g of 2,7-OPOT…..………………………...13-15

Table S3. Cost calculation for the synthesis of 1g of 3,6-OPOT…………..……….......16-17

Figure S8. Estimation of HOMO by PES-AC2 method for (a) spiro-OMeTAD, (b) S-2,7-OPOT and (3) S-3,6-OPOT…………………………………………………………….......18

Figure S9. Estimation of HOMO by PES-AC2 method for (a) 2,7-OPOT and (b) 3,6-OPOT films; (c) UV-vis absorption spectra of 2,7-OPOT and 3,6-OPOT in both solution (line) and film state (dotted line) (d) Energy gap estimated from Tauc plots of 2,7-OPOT and 3,6-OPOT……………………………………………...……………...........................19

Figure S10. Hole-mobility, V_{TFL}, Nt measurements of HTMs by SCLC methods……….......20

Figure S11. The thermogravimetric analysis (TGA) curves of S-2,7-OPOT, S-3,6-OPOT, Spiro-OMeTAD, 2,7-OPOT, and 3,6-OPOT…………………………………………..........20

Figure S12. The DSC curves of spiro-OMeTAD, S-2,7-OPOT and S-3,6-OPOT under N_2 gas at a rate of 10 °C/minutes ………….…………………………………………………....21

Table S4. Thermal properties of HTMs……………………………………………………21

Figure S13. Powder X-ray diffraction (XRD) patterns of S-2,7-OPOT, S-3,6-OPOT, spiro-OMeTAD, 2,7-OPOT and 3,6-OPOT films.………………………...…..…………...22

Figure S14. XPS I 3d core level spectra of perovskite film, spiro-OMeTAD/perovskite, S-2,7-OPOT/perovskite and S-3,6-OPOT/perovskite……………………………...…….23

Figure S15. Dark current measurements of PSCs…………………………......….....24

Table S5. Series resistance (R_s) and shunt resistance (R_{sh}) of PSCs based on spiro-OMeTAD, S-2,7-OPOT and S-3,6-OPOT HTMs..24

Figure S16. The statistical distributions of V_{oc}, J_{sc}, *FF*, and PCE of ten individual devices with HTMs Spiro-OMeTAD, S-2,7-OPOT and S-3,6-OPOT…………………………...25

Device fabrication…………………………..25-26

References………………………………...…………………………………...26-27

Materials and Characterization Methods

All chemicals were purchased from commercial sources and used without further purification. All solvents were carefully dried and freshly distilled according to the standard of laboratory procedures. The reactions carried out under nitrogen atmosphere are mentioned in the scheme. The reactions were monitored by pre-coated TLC and purified by column chromatography using silica gel with mesh size 60-120. Chemical structures were confirmed by the ^{1}H, ^{13}C nuclear magnetic spectroscopy, single crystal X-ray, and high-resolution mass spectrometric analysis (HRMS) techniques. NMR spectra were recorded on a Bruker Avance 400 NMR (400 MHZ) spectrometer. HRMS were obtained by Matrix-assisted laser desorption/ionization technique. Single crystal X-ray diffraction data of 3,6-OPOT was collected at 100 K on a diffractometer. The 3,6-OPOT crystal belongs to the space group of P 21/n. The cell constants are a = 15.3734 Å, b = 17.6300 Å, c = 17.2603 Å, α = 90°, β = 111.365°, γ = 90° and the volume = 4356.6 Å^3. The decomposition temperature T_d were noted as a temperature at which 5% weight loss occur on heating under N_2 atmosphere in a thermogravimetric analyzer. The glass transition temperatures were acquired by differential scanning calorimetric measurements in Perkin Elmer differential scanning calorimeter DSC8000 at the rate of 10 °C/min under N_2. UV-visible absorption spectra were measured in a Varian Gary 300 UV/Vis spectrophotometer. The HOMO values of film samples were estimated from PES-AC2 Reiken Keiki. Energy gap was calculated from Tauc plots. Steady-state photoluminescence (PL) and time-resolved photoluminescence (TRPL) of perovskite and HTM-capped perovskite measurements were conducted at room temperature using a fluorescence spectrophotometer (FLS1000 Photoluminescence Spectrometer, Edinburgh Instruments). The hole mobilities were obtained by a space-charge limited current (SCLC) method, which was carried out by using FTO/PEDOT:PSS/$MAPbI_3$/HTM/Ag. PEDOT:PSS (filtered through a 0.45 μm PVDF filter) layer was spin-coated (4000 rpm, 30 seconds) on top of pre-cleaned FTO substrates and annealed at 150 °C for 10 min. All other technical aspects are similar to the full device fabrication process. The hole mobility (μ) data were then precisely analyzed according to the Mott–Gurney law[1]:

$$J = \frac{9}{8}\varepsilon_0\, \varepsilon_r \mu \frac{V^2}{L^3} \quad \text{[Eq. 1]}$$

Where J is the current density, ε_0 is the vacuum permittivity, ε_r is the relative permittivity constant of the active layer, L is the thickness of the active layer, and V is the applied voltage. The defect density was calculated by $N_t = (2\varepsilon\varepsilon_0 V_{TFL})/(qL^2)$, where q is the elementary charge and V_{TFL} is the trap filled limit voltage.[2]

A PHI-5000 Versa Probe X-ray photoelectron spectrometer was used to obtain the Pb 4f and I 3d core level XPS spectra. The surface morphology of the films was characterized by field emission scanning electron microscopy (FE-SEM, ULTRA PLUS, Carl Zeiss). Water contact angle was measured with PSC-1000 B image contact angle measuring instrument (Pentad Scientific Corroboration, Taiwan) having FTA-22AUC03 CCD camera with SBL-3030-B high bright tech LED.

The solar cell efficiencies were evaluated at ambient conditions using a Keithley 2400 digital source meter from Class AAA Solar Simulator (SAN-EI ELECTRIC Co., Ltd. Japan) with a simulated AM 1.5G irradiation of 100 $mWcm^{-2}$ (1 sun). A monocrystalline silicon reference cell (Oriel Instruments, Newport 91150 V) was used for calibration of the light intensity. The incident photon to current conversion efficiency (IPCE) spectra were measured in air using a commercial IPCE setup (LiveStrong optoelectronics Co., Ltd., Taiwan-Model No.: LSQE-N) with a broad band light source, grating monochromator: 350 nm to 1100 nm scanning range, spectral bandwidth < 5 nm and probe light modulation frequency from 1 Hz to 100 Hz. The IPCE was analyzed under a light intensity of 100 mW cm^{-2} (AM 1.5G). Prior to the IPCE measurement, a monocrystalline silicon diode is used as a standard reference for calibration. The $J-V$ and IPCE measurements were conducted with a 0.04 cm^2 active area mask.

Synthetic Schemes and Experimental Procedures

Compounds M_2,[3] M_3,[4] M_6,[5] M_7,[5] M_8,[6] and M_9[4] were synthesized according to the previous literature procedures in **Scheme S1**.

Scheme S1. Synthesis of 2,7-OPOT and 3,6-OPOT.

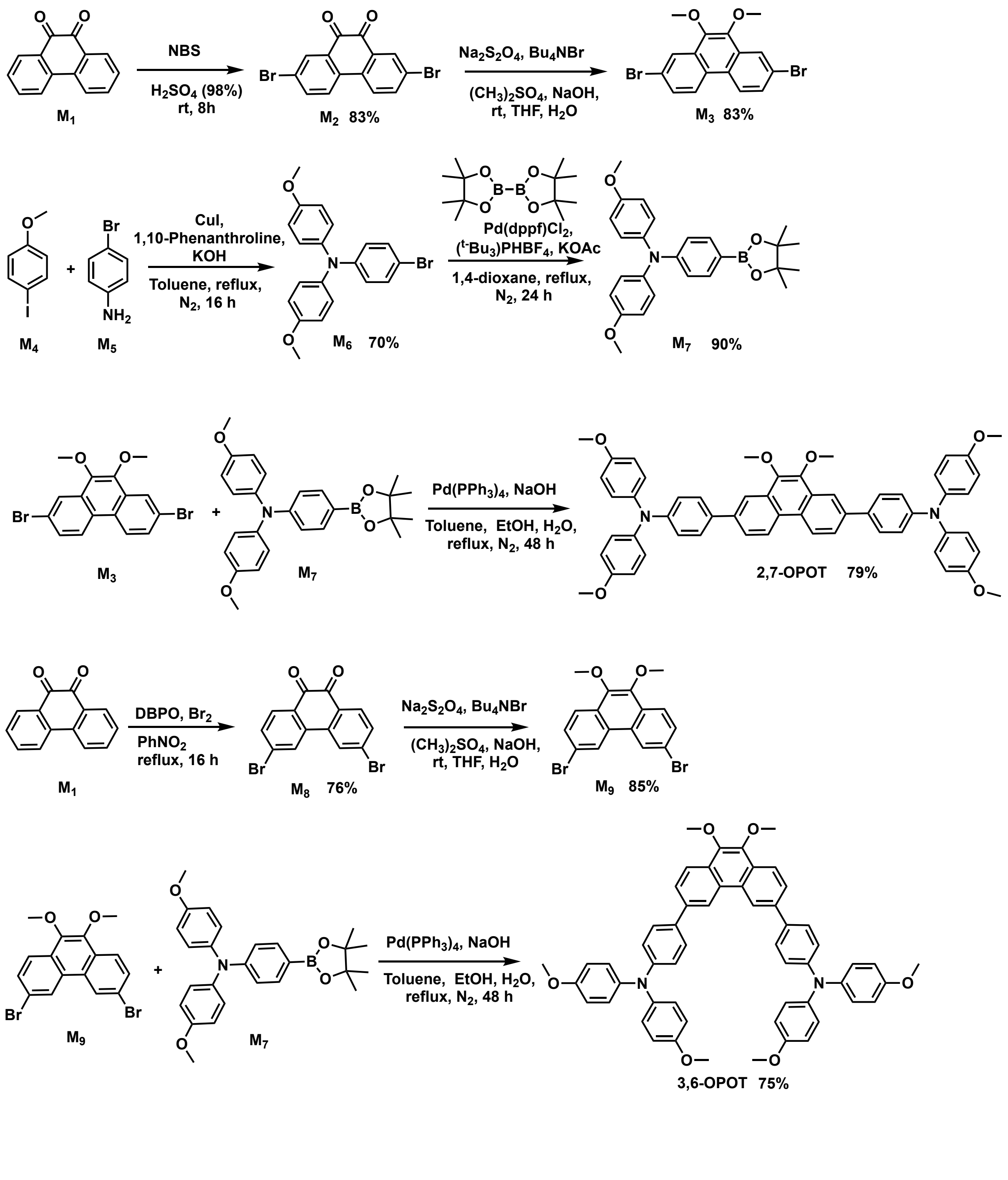

Synthesis of 4,4'-(9,10-dimethoxyphenanthrene-2,7-diyl)bis(*N*,*N*-bis(4-methoxyphenyl)aniline) (2,7-OPOT)

In an oven-dried Schlenk flask, M3 (0.300 g, 0.757 mmol), M7 (0.977 g, 2.27 mmol), $Pd(PPh_3)_4$ (0.175 g, 0.151 mmol), and NaOH (0.182 g, 4.54 mmol) were weighed and degassed three times. A mixture of solvents containing toluene, ethanol and deionized water with 12 mL, 8 mL and 4 mL (3:2:1, v/v), respectively, priorly purged by N_2, was transferred to the Schlenk flask. The solution was allowed to reflux for 48 h under the N_2 atmosphere. After completion of the reaction, solvent was evaporated and the residual was extracted with 100 mL of ethyl acetate. The crude was subjected to a silica column chromatographic separation using 15% ethyl acetate/hexane eluent to obtain a pale greenish-yellow solid with a yield of 79%.

^{1}H NMR (400 MHz, DMSO-d6) δ (ppm): 8.79 (d, *J* = 8.8 Hz, 2H), 8.28 (d, *J* = 1.6 Hz, 2H), 7.88 (dd, *J* = 8.8 Hz, and *J* = 2 Hz, 2H), 7.69 (d, *J* = 8.8 Hz, 4H), 7.09 (d, *J*= 8.8 Hz, 8H), 6.95 (d, *J*= 8.8 Hz, 8H), 6.91(d, *J* = 8.4 Hz, 4H), 4.05 (s, 6H), 3.76 (s, 12H). ^{13}C NMR (100 MHz, $CDCl_3$) δ (ppm): 156.09, 148.48, 144.54, 141.03, 139.11, 132.97, 129.42, 127.96, 126, 80, 124.83, 123.30, 120.94, 119.32, 114.90, 61.14, 55.66. MALDI-HRMS (M^+) calcd for $C_{56}H_{48}N_2O_6$: 844.3512; found: 844.3536.

Synthesis of 4,4'-(9,10-dimethoxyphenanthrene-3,6-diyl)bis(*N*,*N*-bis(4-methoxyphenyl)aniline) (3,6-OPOT)

Compound 3,6-OPOT was synthesized by following a similar procedure for 2,7-OPOT using M9 (0.400 g, 1.01 mmol), M7 (1.31 g, 3.03 mmol), $Pd(PPh_3)_4$ (0.231 g, 0.200 mmol), and NaOH (0.240 g, 0.600 mmol). After purification, a pale-yellow solid (75%) was obtained.

^{1}H NMR (400 MHz, DMSO-d6) δ (ppm): 9.04 (s, 2H), 8.18 (d, *J* = 8.8 Hz, 2H), 7.93(dd, *J* = 8.8 Hz, *J* = 1.2 Hz, 2H), 7.80 (d, *J* = 8.8 Hz, 4H), 7.08 (d, *J* = 8.8 Hz, 8H), 6.95 (d, *J* = 8.8 Hz, 8H), 6.91(d, *J* = 8.8 Hz, 4H), 4.04(s, 6H), 3.76 (s, 12H). ^{13}C NMR (100 MHz, $CDCl_3$) δ (ppm): 156.08, 148.44, 143.89, 141.04, 138.47, 133.37, 129.17, 128.09, 126.78, 126.02, 122.80, 121.03, 120.38, 114.90, 61.18, 55.66. MALDI-HRMS (M^+) calcd for $C_{56}H_{48}N_2O_6$: 844.3512; found: 844.3515.

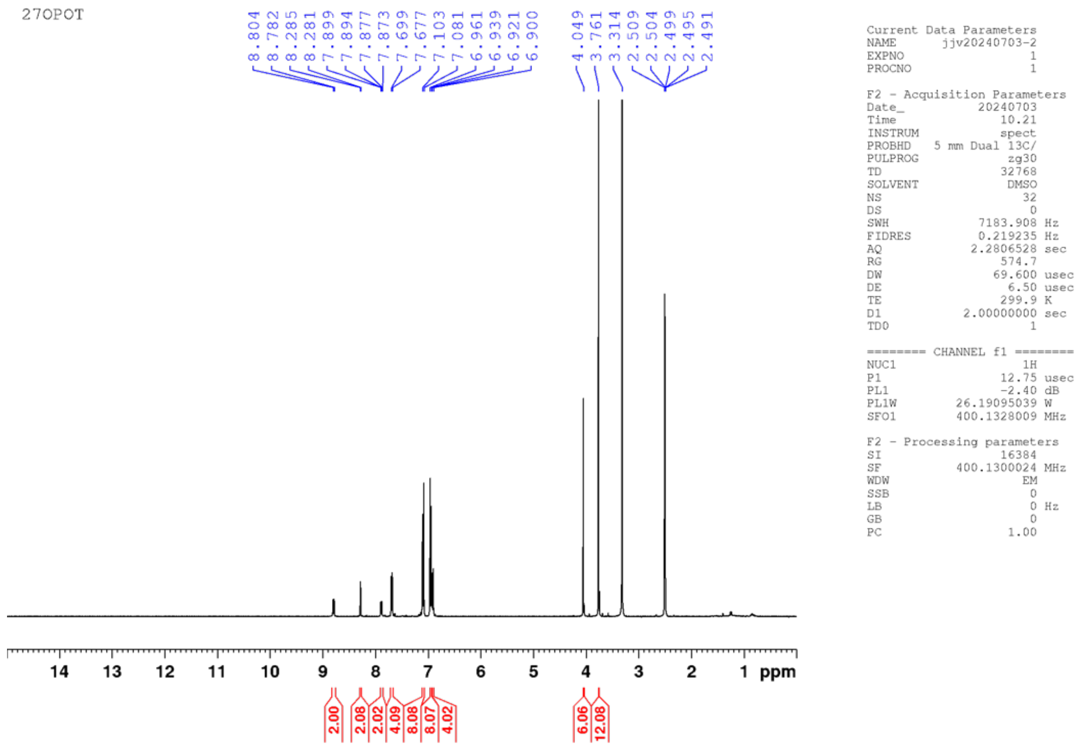

Figure S1. ^{1}H NMR of 2,7-OPOT (400 MHz, DMSO-d_6).

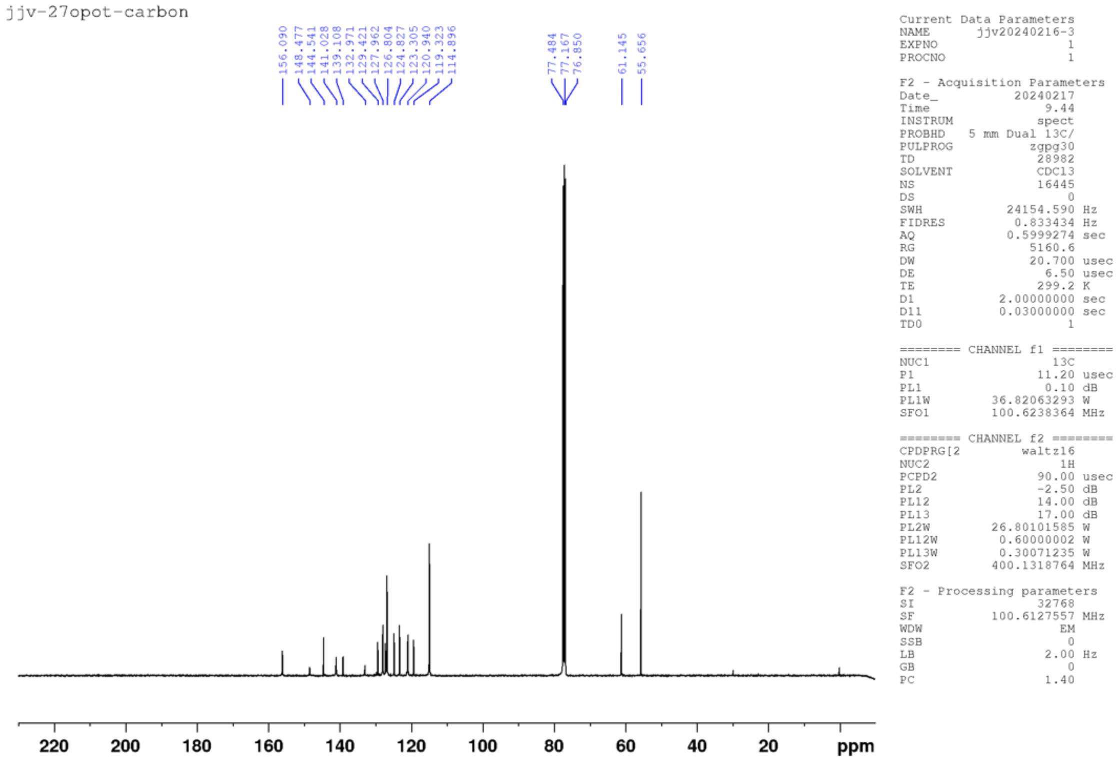

Figure S2. ^{13}C NMR of 2,7-OPOT (100 MHz, $CDCl_3$).

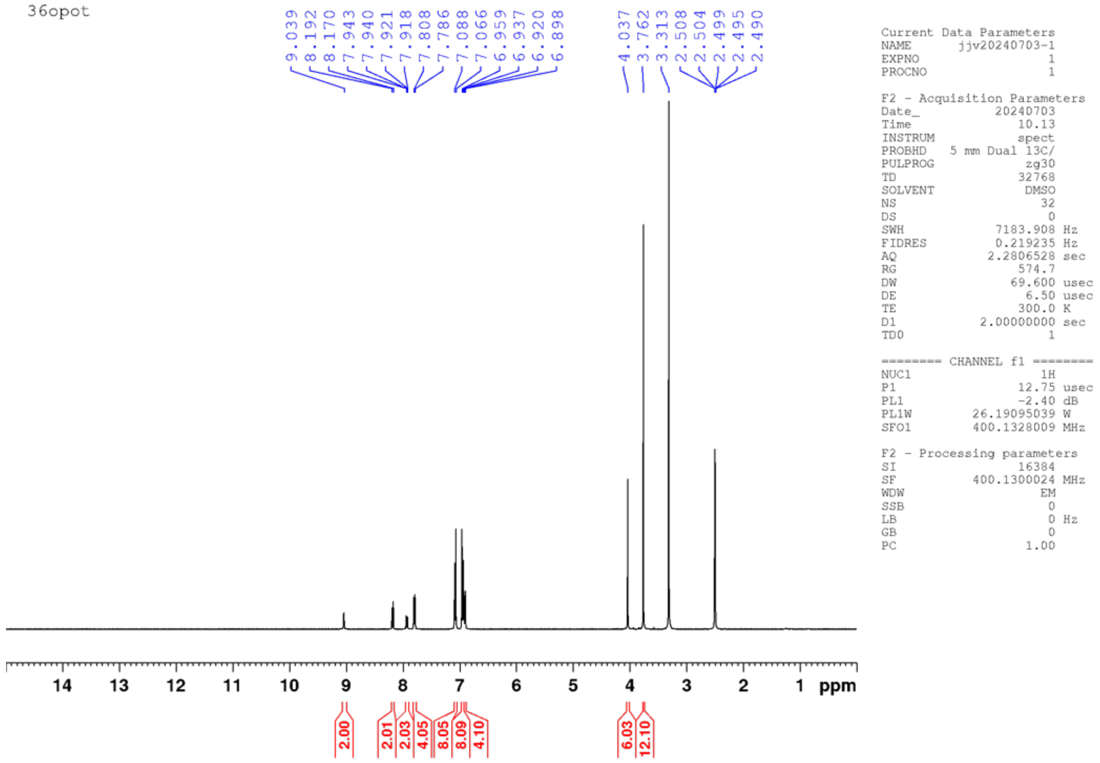

Figure S3. ^{1}H NMR of 3,6-OPOT (400 MHz, DMSO-d_6).

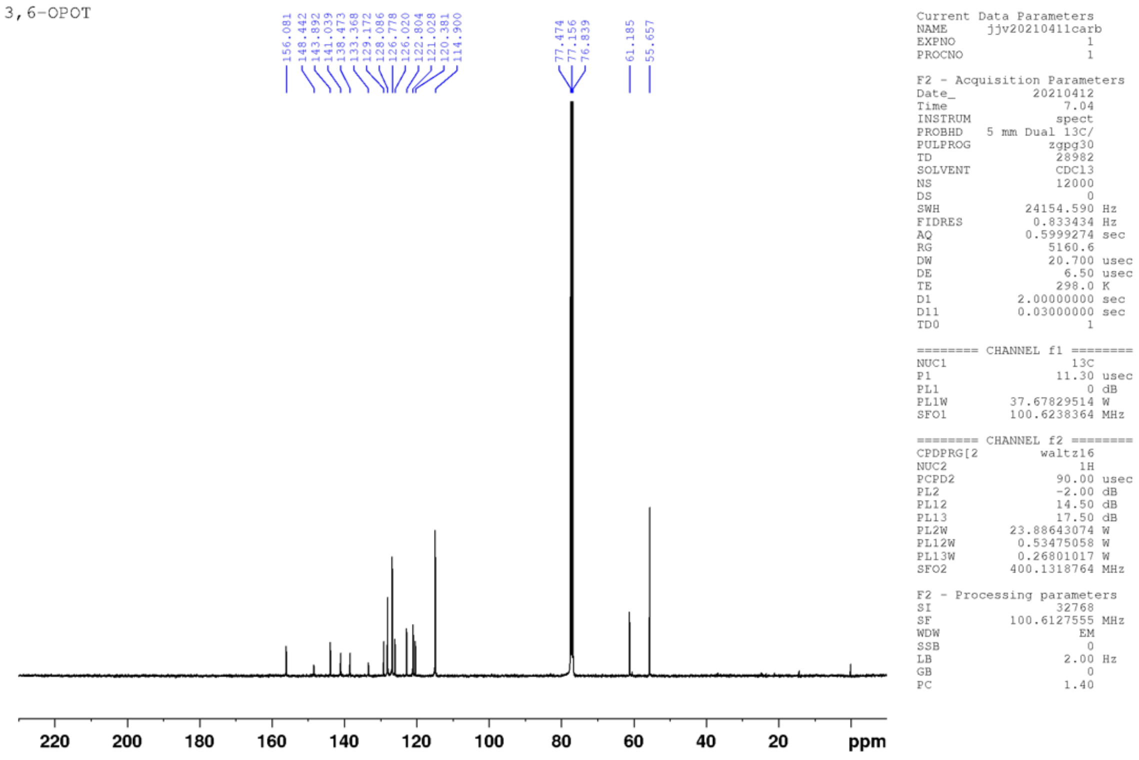

Figure S4. ^{13}C NMR of 3,6-OPOT (100 MHz, $CDCl_3$).

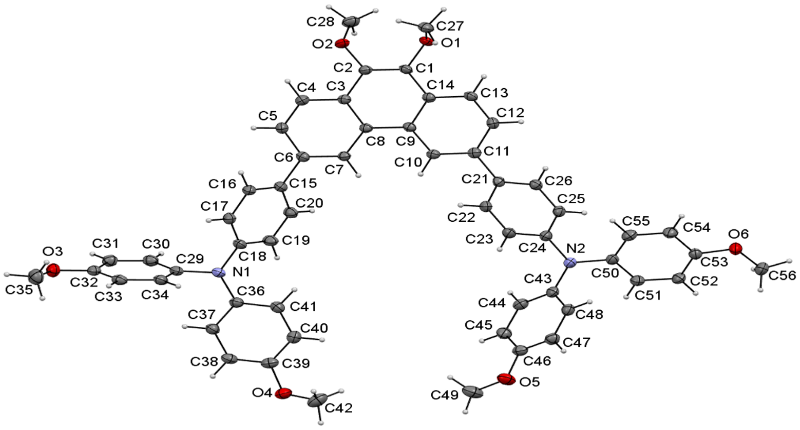

Figure S5. Single-crystal structure of 3,6-OPOT.

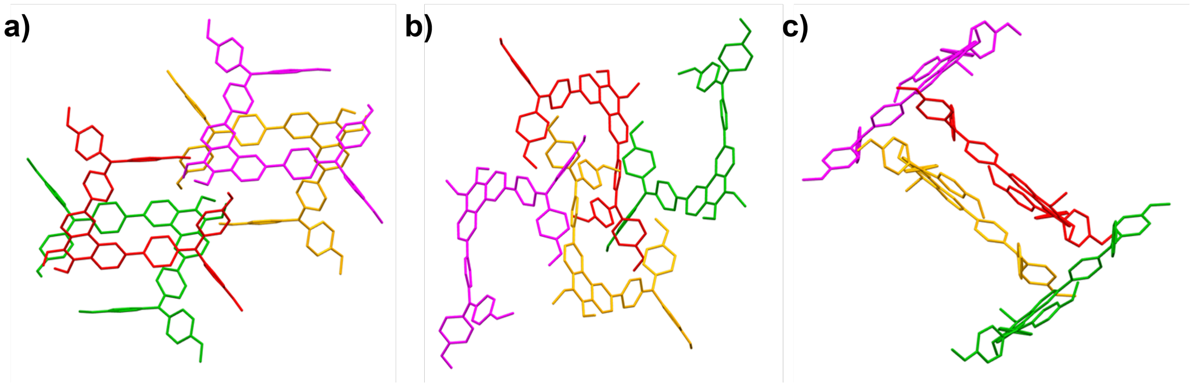

Figure S6. (a-c) The crystal packing of 3,6-OPOT along axis a, b and c.

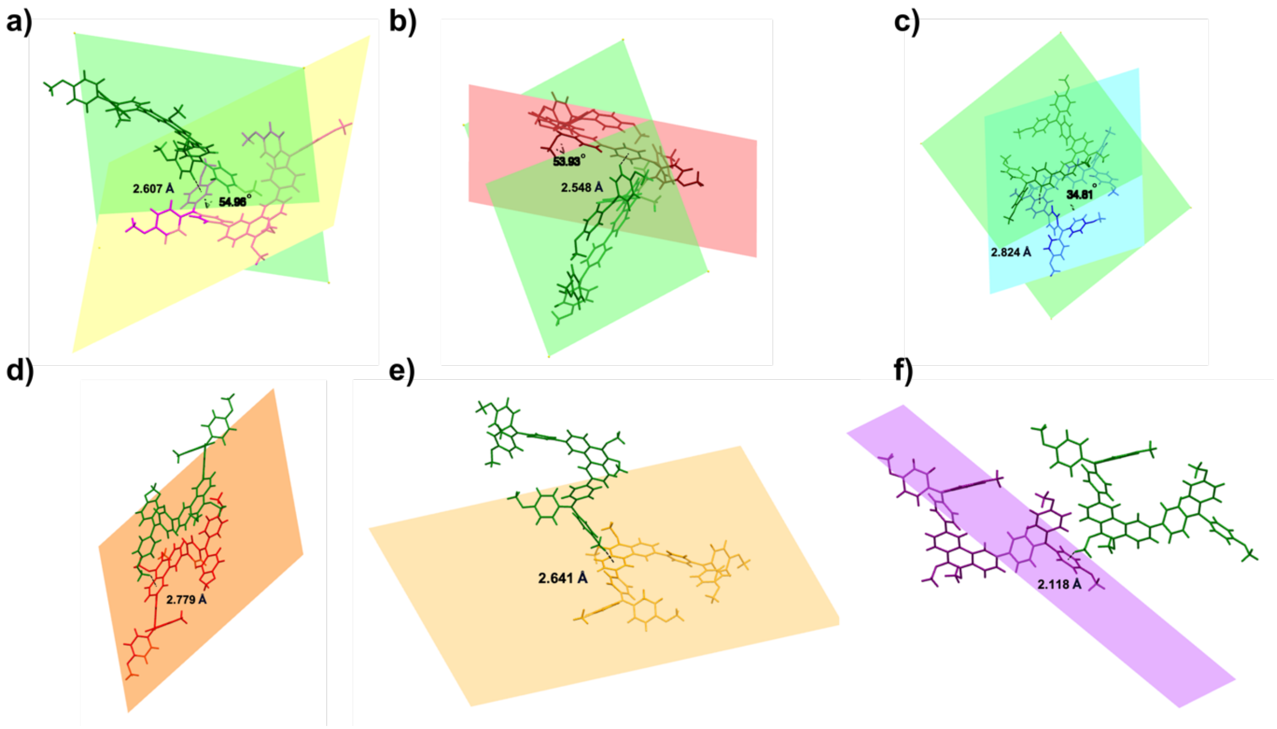

Figure S7. CH-π interactions extracted from single crystal analysis of 3,6-OPOT with distance and dihedral angle of a) 2.607 Å, 54. 96° b) 2.548 Å, 53. 93° c) 2.824 Å, 34.81° d) 2.779 Å e) 2.641 Å f) 2.118 Å.

Table S1 Crystal data and structure refinement for 3,6-OPOT.

Empirical formula	C56 H48 N2 O6	
Formula weight	844.96	
Temperature	100.0(2) K	
Wavelength	0.71073 Å	
Crystal system	Monoclinic	
Space group	P 21/n	
Unit cell dimensions	a = 15.3734(5) Å	a= 90°.
	b = 17.6300(5) Å	b= 111.3650(10)°.
	c = 17.2603(6) Å	g = 90°.
Volume	4356.6(2) $Å^3$	
Z	4	
Density (calculated)	1.288 Mg/m^3	
Absorption coefficient	0.083 mm^{-1}	
F(000)	1784	

Crystal size	0.255 x 0.193 x 0.140 mm^3
Theta range for data collection	1.714 to 27.102°.
Index ranges	-19<=h<=19, -22<=k<=22, -22<=l<=22
Reflections collected	130876
Independent reflections	9611 [R(int) = 0.1073]
Completeness to theta = 25.242°	99.9%
Absorption correction	Numerical
Max. and min. transmission	1 and 0.9467
Refinement method	Full-matrix least-squares on F^2
Data / restraints / parameters	9611 / 0 / 583
Goodness-of-fit on F^2	1.020
Final R indices [I>2sigma(I)]	R1 = 0.0442, wR2 = 0.1046
R indices (all data)	R1 = 0.0740, wR2 = 0.1208
Extinction coefficient	n/a
Largest diff. peak and hole	0.299 and -0.286 e.$Å^{-3}$

Table S2. Material cost for the synthesis of 1g of 2,7-OPOT

Step 1.

Reagent	Amount (g)	Amount (mL)	Price \$/g or \$/mL	Price of chemical used (\$)	Total cost (\$)
Phenanthrene-9,10-dione	7	-	1.35	9.45	14.5008\$/10.2129 g or **1.4198\$/g**
NBS	12.86	-	0.18	2.3148	
H_2SO_4	-	190	0.0144	2.7360	
Product = **2,7-dibromophenanthrene-9,10-dione**					

Step 2.

Reagent	Amount (g)	Amount (mL)	Price \$/g \$/mL	Price of chemical used (\$)	Total cost (\$)
2,7-dibromophenanthrene-9,10-dione	2.5	-	1.4198	3.5495	7.4416\$/2.2454 g or **3.3141\$/g**
$Na_2S_2O_4$	3.5670	-	0.063	0.2247	
Bu_4NBr	0.7040	-	0.66	0.4646	
$(CH_3)_2SO_4$	-	3.36	0.0225	0.0756	
NaOH	3.56	-	0.021	0.0747	
THF	-	30	0.0105	0.315	
H_2O	-	50	0	0	
Silica gel	50	-	0.01	0.5	
n-Hexane	-	265	0.0067	1.7755	
Ethylacetate	-	140	0.0033	0.462	
Product = **2,7-dibromo-9,10-dimethoxyphenanthrene**					

Step 3.

Reagent	Amount (g)	Amount (mL)	Price \$/g \$/mL	Price of chemical used (\$)	Total cost (\$)

1-Iodo-4-methoxybenzene	8.1628	-	1.11	9.0607	13.1728$/3.1273g or **4.2121$/g**
4-Bromoaniline	2	-	0.324	0.648	
CuI	0.7966	-	0.396	0.3154	
1,10-Phenanthroline	0.0774	-	3.15	0.2438	
KOH	5.21	-	0.0228	0.1187	
Toluene	-	75	0.00375	0.2812	
Silica gel	50	-	0.01	0.5	
n-Hexane	-	250	0.0067	1.675	
Ethylacetate	-	100	0.0033	0.33	
Product = **4-bromo-*N*,*N*-bis(4-methoxyphenyl)aniline**					

Step 4.

Reagent	Amount (g)	Amount (mL)	Price $/g $/mL	Price of chemical used	Total cost ($)
4-bromo-*N*,*N*-bis(4-methoxyphenyl)aniline	1.5	-	4.2121	6.3181	21.3514$/1.5153g or **14.0905$/g**
4,4,4',4',5,5,5',5'-octamethyl-2,2'-bi(1,3,2-dioxaborolane)	1.486	-	0.9	1.3374	
Pd(dppf)Cl_2	0.0142	-	54	0.7668	
($^{t-}Bu_3$)$PHBF_4$	0.0226	-	18	0.4068	
KOAc	1.1973	-	0.108	0.1293	
1,4-dioxane	-	50	0.162	8.1	
Silica gel	30	-	0.01	0.3	
n-Hexane	-	495	0.0067	3.3165	
Ethylacetate	-	205	0.0033	0.6765	
Product = **4-methoxy-*N*-(4-methoxyphenyl)-*N*-(4-(4,4,5,5-tetramethyl-1,3,2-dioxaborolan-2-yl)phenyl)aniline**					

Step 5.

Reagent	Amount (g)	Amount (mL)	Price $/g or $/mL	Price of chemical used ($)	Total cost ($)
2,7-dibromo-9,10-dimethoxyphenanthrene	0.300	-	3.3141	0.9942	23.8673/0.51g
4-methoxy-*N*-(4-methoxyphenyl)-*N*-(4-(4,4,5,5-tetramethyl-1,3,2-dioxaborolan-2-yl)phenyl)aniline	0.9770	-	14.0905	13.7664	or<br><br>**46.7986 $/g**
$Pd(PPh_3)_4$	0.1750	-	30	5.25	
NaOH	0.1817	-	0.021	0.0038	
Toluene	-	12	0.0037	0.0444	
Ethanol	-	8	0.0225	0.18	
H_2O	-	4	0	0	
Silica gel	25	-	0.01	0.25	
n-Hexane	-	455	0.0067	3.0485	
Ethylacetate	-	100	0.0033	0.33	
Product = **4,4'-(9,10-dimethoxyphenanthrene-2,7-diyl)bis(*N*,*N*-bis(4-methoxyphenyl)aniline)**					

Table S3. Material cost for the synthesis of 1g of 3,6-OPOT.

Step 1.

Reagent	Amount (g)	Amount (mL)	Price $/g $/mL	Price of chemical used	Total cost ($)
phenanthrene-9,10-dione	10.2	-	1.35	13.77	18.5052$/13.6266g or **1.3580$/g**
Dibenzoylperoxide	0.975	-	1.152	1.1232	
Br_2	-	5.1	0.054	0.2754	
$PhNO_2$	-	62	0.0430	2.6660	
n-Hexane	-	100	0.0067	0.6700	
Product = **3,6-dibromophenanthrene-9,10-dione**					

Step 2.

Reagent	Amount (g)	Amount (mL)	Price $/g $/mL	Price of chemical used ($)	Total cost ($)
3,6-dibromophenanthrene-9,10-dione	2.5	-	1.3580	3.395	7.2871$/2.2995g or **3.1689$/g**
$Na_2S_2O_4$	3.5670	-	0.063	0.2247	
Bu_4NBr	0.7040	-	0.66	0.4646	
$(CH_3)_2SO_4$	-	3.36	0.0225	0.0756	
NaOH	3.56	-	0.021	0.0747	
THF	-	30	0.0105	0.315	
H_2O	-	50	0	0	
Silica	50	-	0.01	0.5	
n-Hexane	-	265	0.0067	1.7755	
Ethylacetate	-	140	0.0033	0.462	
Product = **3,6-dibromo-9,10-dimethoxyphenanthrene**					

Step 3.

Reagent	Amount (g)	Amount (mL)	Price $/g $/mL	Price of chemical used ($)	Total cost ($)
3,6-dibromo-9,10-dimethoxyphenanthrene	0.4	-	3.1689	1.2675	30.5119 $/0.64 g
4-methoxy-*N*-(4-methoxyphenyl)-*N*-(4-(4,4,5,5-tetramethyl-1,3,2-dioxaborolan-2-yl)phenyl)aniline	1.3068	-	14.0905	18.4134	
$Pd(PPh_3)_4$	0.2313	-	30	6.939	
NaOH	0.24	-	0.021	0.0050	
Toluene	-	12	0.00375	0.045	
Ethanol	-	8	0.0225	0.18	
H_2O	-	4	0	0	
Silica	30	-	0.01	0.3	
n-Hexane	-	455	0.0067	3.0485	
Ethylacetate	-	95	0.0033	0.3135	
Product = **4,4'-(9,10-dimethoxyphenanthrene-3,6-diyl)bis(*N*,*N*-bis(4-methoxyphenyl)aniline)**					**47.6748 $/g**

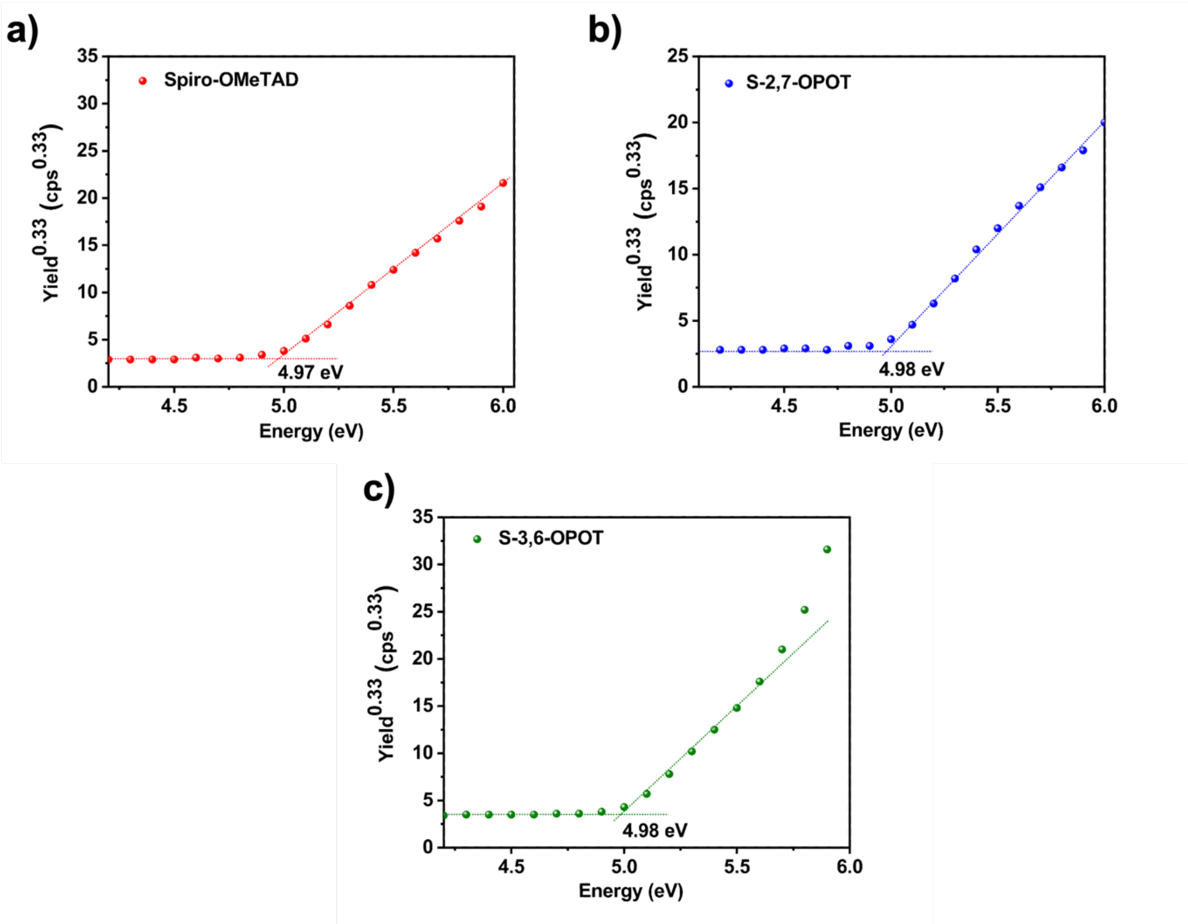

Figure S8. Estimation of HOMOs by PESA-AC2 method for (a) spiro-OMeTAD, (b) 2,7-OPOT and (c) 3,6-OPOT films.

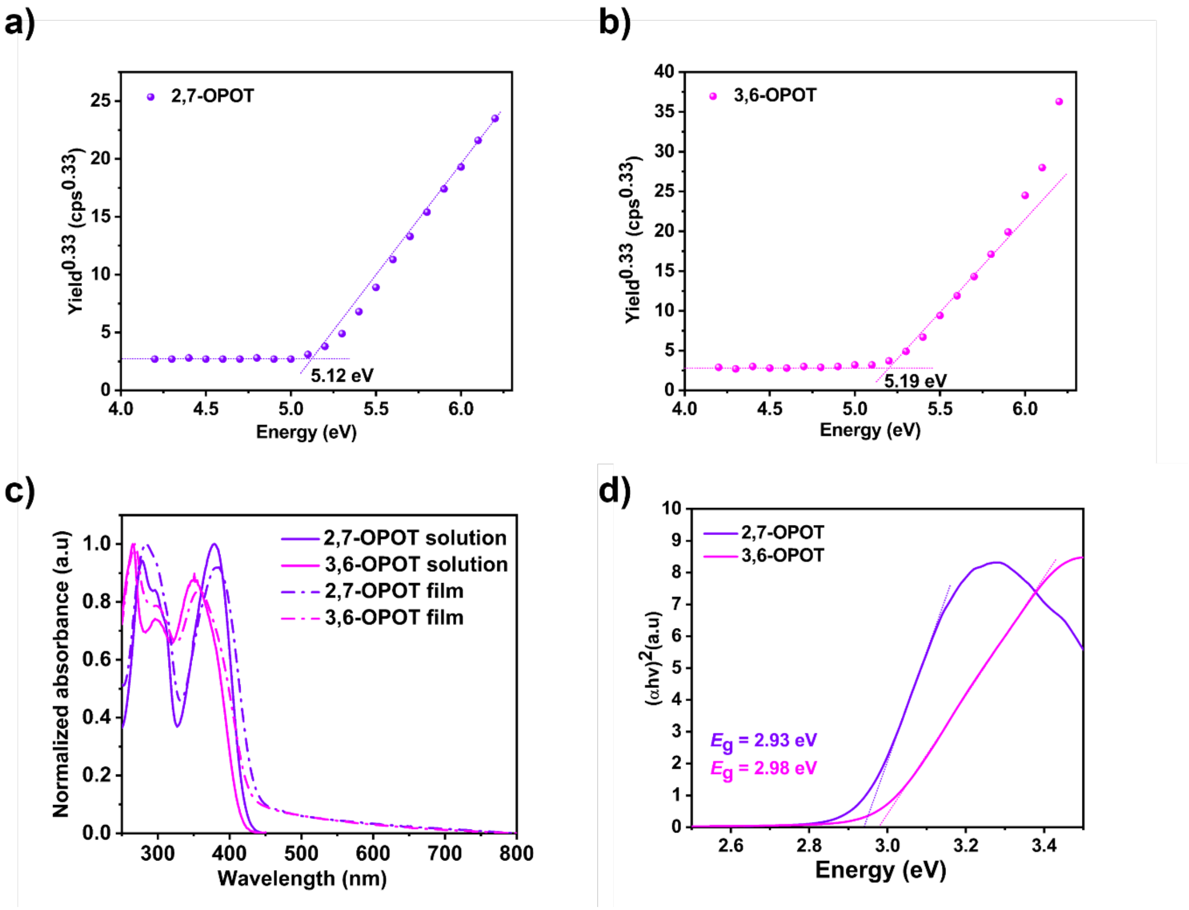

Figure S9. Estimation of HOMO by PESA-AC2 method for (a) 2,7-OPOT and (b) 3,6-OPOT films; (c) UV-vis absorption spectra of 2,7-OPOT and 3,6-OPOT in both solution (line) and film state (dotted line). (d) Energy gap estimated from Tauc plots of 2,7-OPOT and 3,6-OPOT.

.

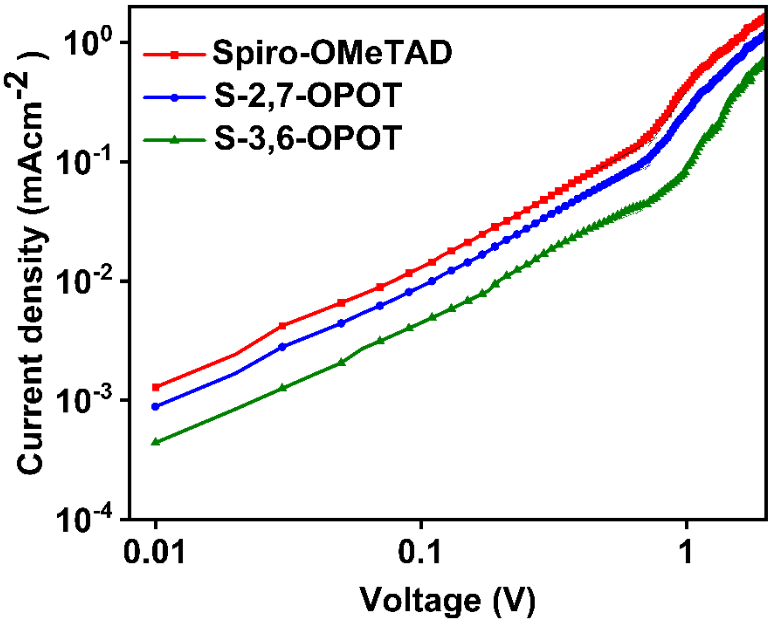

Figure S10. Hole-mobility, V_{TFL} and N_t measurements of composite HTMs and spiro-OMeTAD by SCLC method.

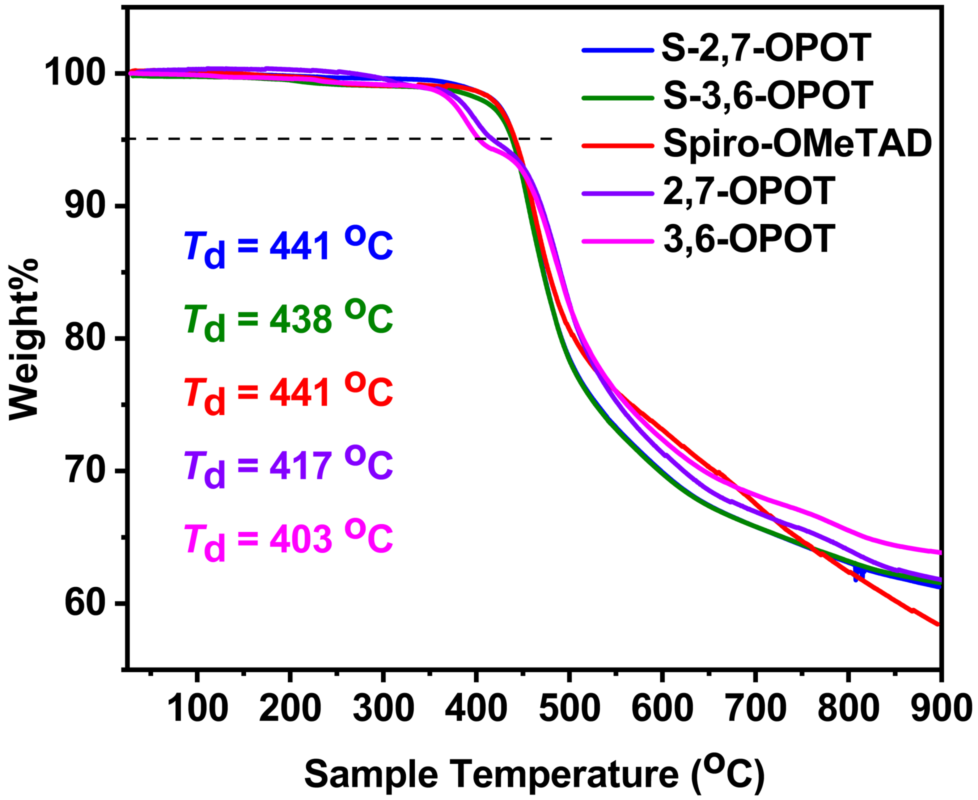

Figure S11. The thermogravimetric analysis (TGA) curves of S-2,7-OPOT, S-3,6-OPOT, spiro-OMeTAD, 2,7-OPOT, and 3,6-OPOT under N_2.

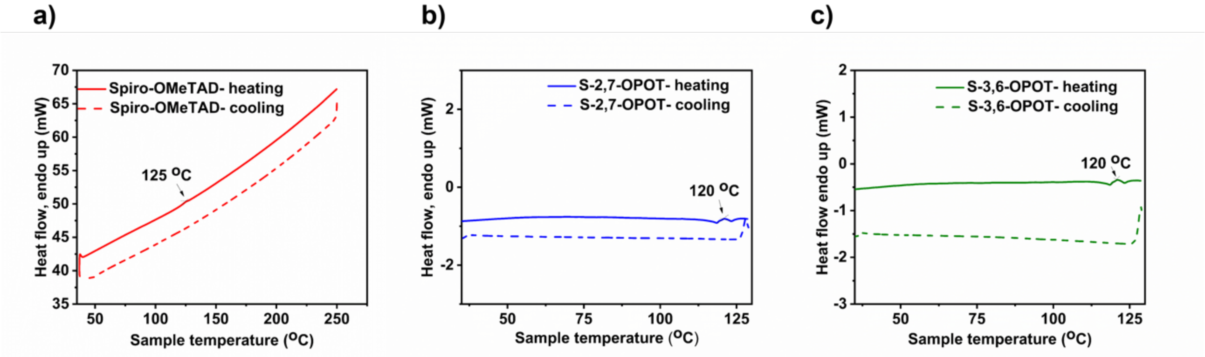

Figure S12. The DSC curves of spiro-OMeTAD, S-2,7-OPOT and S-3,6-OPOT at a rate of 10 °C/minutes under N_2 gas.

Table S4. Thermal Properties of HTMs.

HTM	T_d[a] [°C]	T_g[b] [°C]
S-2,7-OPOT	441	125
S-3,6-OPOT	438	120
spiro-OMeTAD	441	120

[a]Decomposition temperature measured under N_2. [b]Glass transition temperature measure under N_2 at the rate of 10 °C/minute.

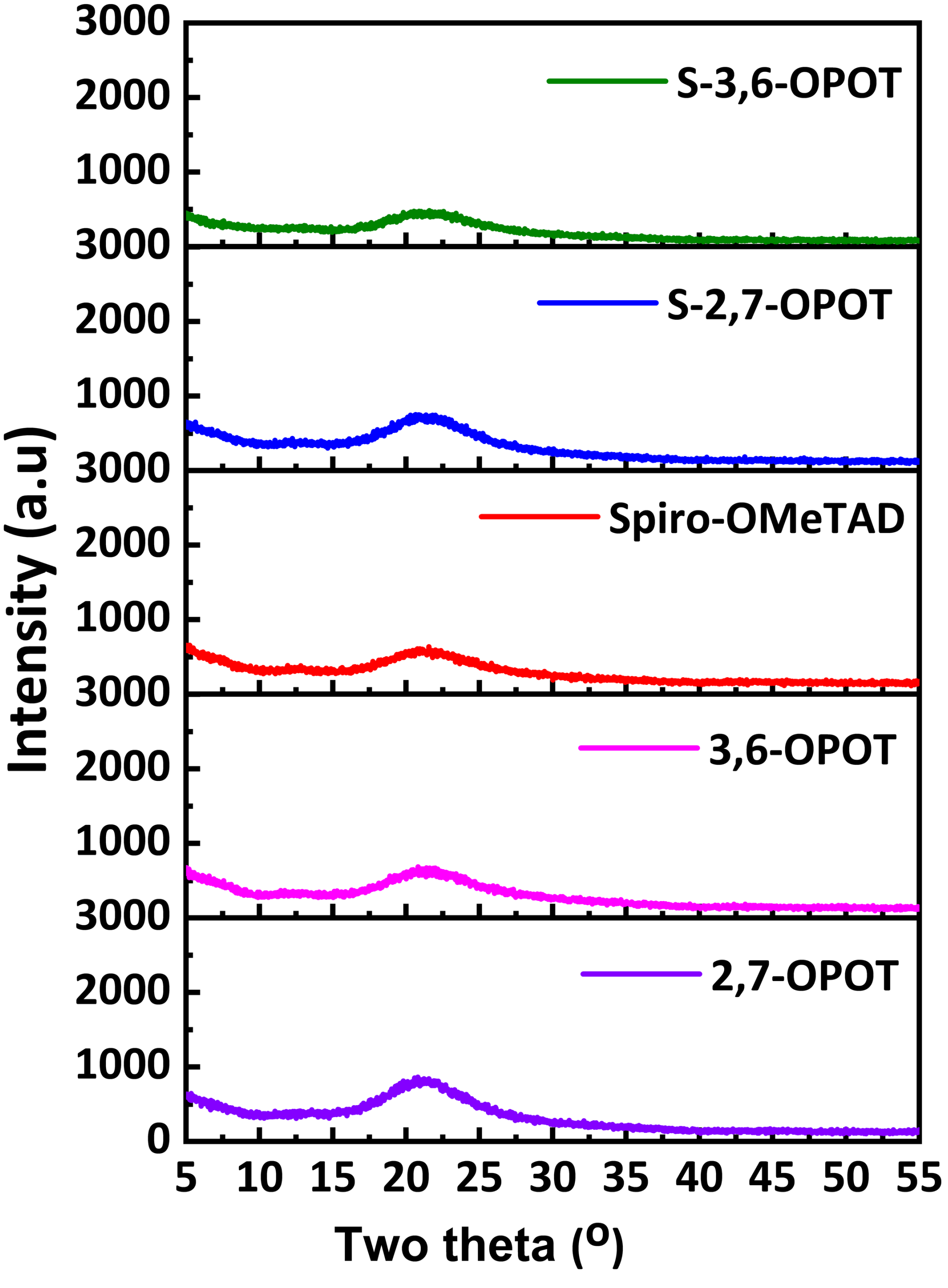

Figure S13. X-ray diffraction (XRD) patterns of 2,7-OPOT, 3,6-OPOT, spiro-OMeTAD, S-2,7-OPOT, S-3,6-OPOT films.

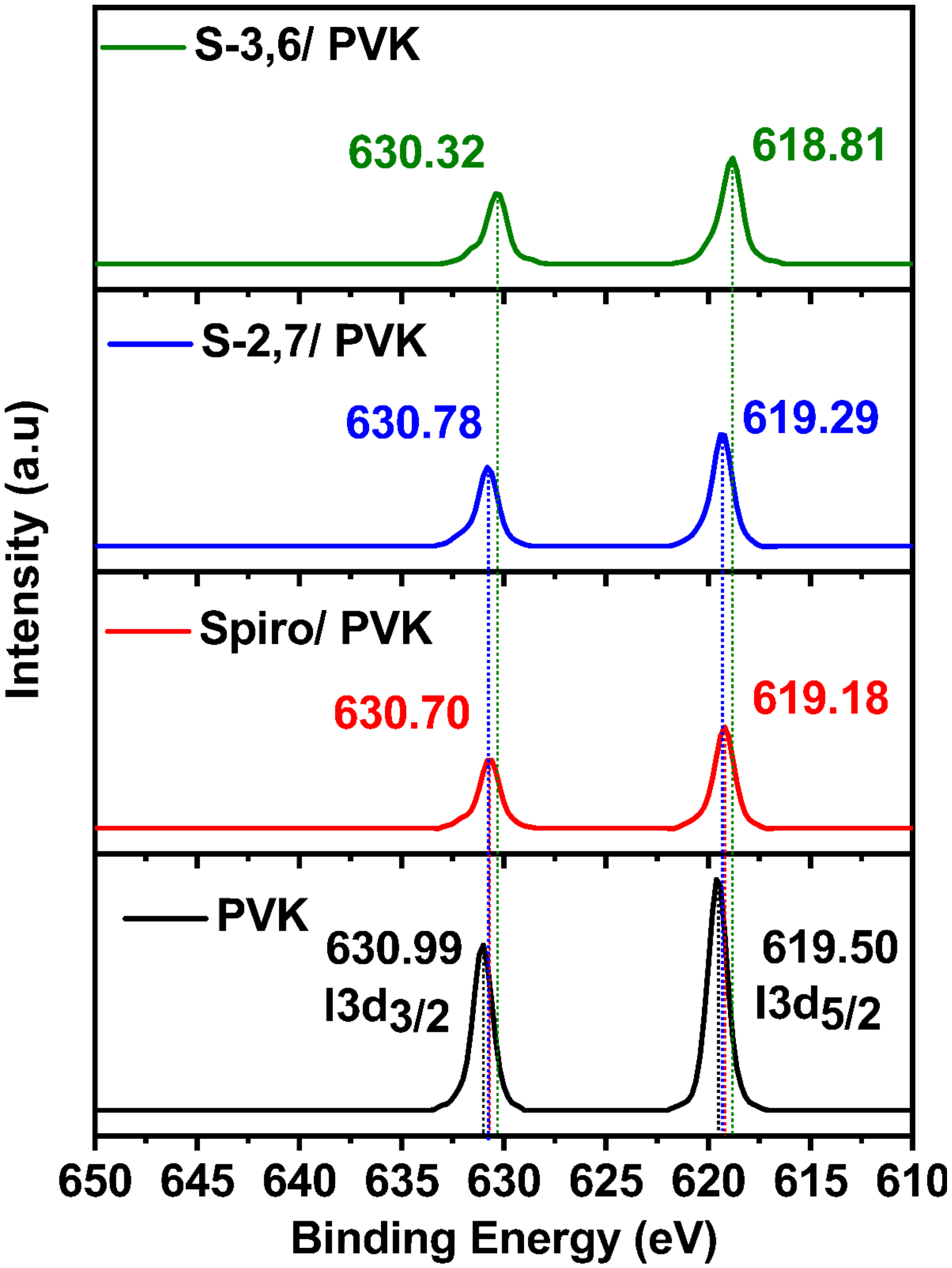

Figure S14. XPS I 3d core level spectra of perovskite film, spiro-OMeTAD/perovskite, S-2,7-OPOT/perovskite and S-3,6-OPOT/perovskite.

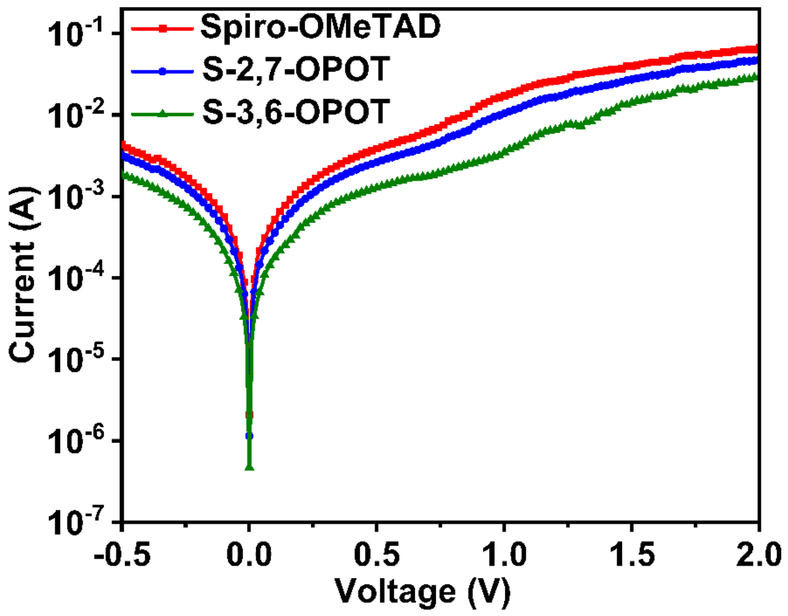

Figure S15. Dark current measurements of PSCs.

Table S5. R_s and R_{sh} of PSCs based on spiro-OMeTAD, S-2,7-OPOT and S-3,6-OPOT HTMs

HTM	R_s [Ω cm^2]	R_{sh} [kΩ cm^2]
spiro-OMeTAD	52.9	12.8
S-2,7-OPOT	43.7	18.2
S-3,6-OPOT	42.3	18.5

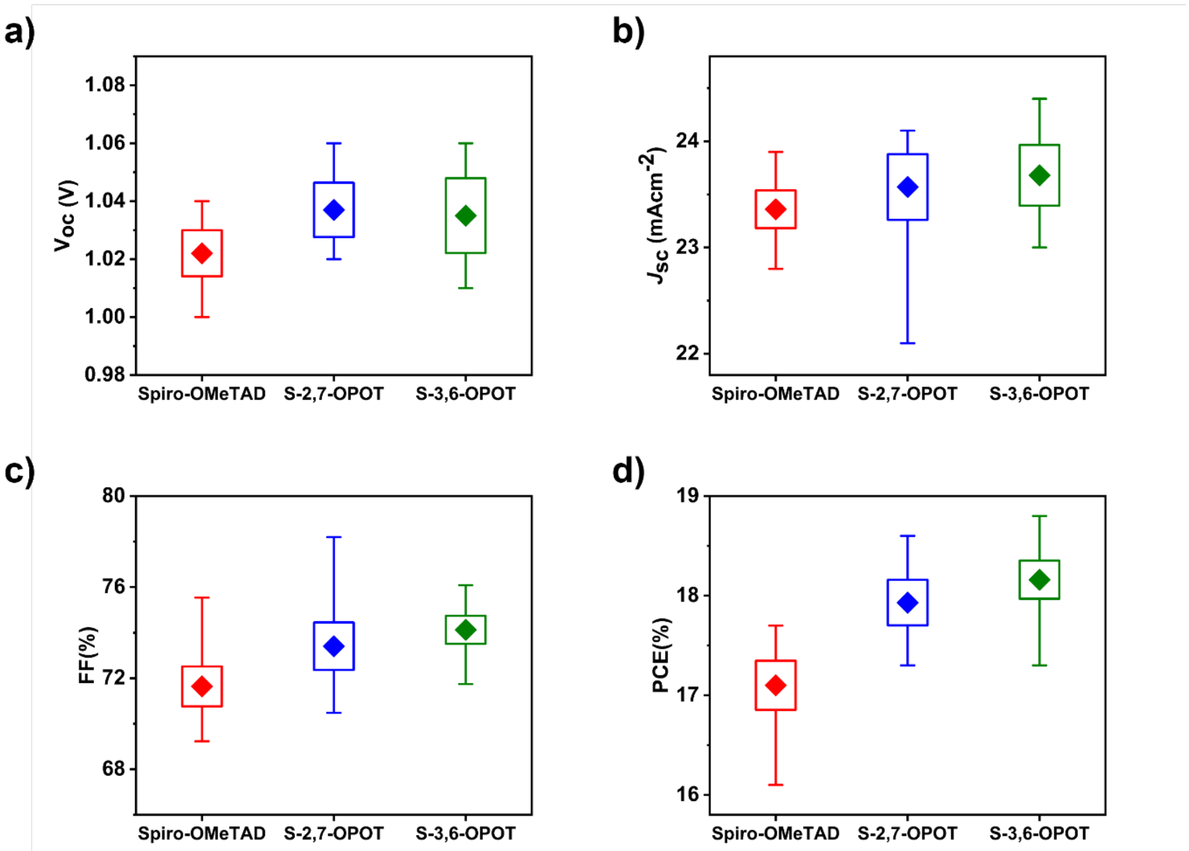

Figure S16. The statistical distributions of a) V_{oc}, b) J_{sc}, c) FF, and d) PCE of ten individual devices with HTMs Spiro-OMeTAD, S-2,7-OPOT and S-3,6-OPOT.

Device Fabrication

The fabrication PSC was carried out following the previously reported procedure.[7] The FTO substrates were etched with zinc powder and 2 M of HCl, and then cleaned by a detergent, deionized water, acetone and isopropanol with sonication for 15 minutes each, respectively. Before device fabrication, the cleaned FTO substrates were dried under a flow of N_2 gas and treated with UV ozone for 20 minutes. The compact TiO_2 (cp-TiO_2) solution consisted of titanium diisopropoxide bis(acetylacetonate) (75 wt% in isopropanol) was diluted in butanol (1:18, v/v). The corresponding solution was then passed through 0.22 µm polyvinylidene fluoride (PVDF) filter before use. The filtered cp-TiO_2 precursor solution was spin-coated on the UV ozone pre-cleaned FTO substrate at 2000 rpm for 40 seconds. The resulting film was

annealed at 100, 175, 225, 375 and 480 °C for 5, 5, 5, 15 and 30 minutes, respectively. After cooling the substrate to room temperature and treated with UV ozone for 15 minutes, the mesoporous TiO_2 (mp-TiO_2) paste dissolved in absolute ethanol (150 mg in 1 mL) was spin-coated on cp-TiO_2 layer at 5000 rpm for 30 seconds. Then, the film was annealed in the same way as the cp-TiO_2 film. For the deposition of a perovskite active layer, the FTO/TiO_2 substrates were transferred into a glovebox filled with nitrogen. The perovskite precursor solution was prepared by dissolving 461 mg of PbI_2, and 159 mg of CH_3NH_3I, in of DMF/DMSO (9:1, v/v ratio) and stirred at room temperature for 6 h, and then spin-coated via a one-step deposition technique at 4000 rpm for 25 seconds. A total of 150 μL of anhydrous chlorobenzene was rapidly dripped on top of the substrate at the 10th seconds. The perovskite-spin-coated substrates were annealed at 100 °C for 5 minutes. Subsequently, a hole-transporting material was deposited at 4000 rpm for 30 seconds. For the control device, a 40 mg of spiro-OMeTAD was dissolved in 0.5 mL of chlorobenzene with dopant 7.5 μL LITFSI (prepared by dissolving 50 mg in 100 μL of ACN) and TBP (14.5 μL). On the other hand, the composite HTM solution of S-2,7-OPOT and S-3,6-OPOT was prepared by combining in a 3:1 v/v ratio of spiro-OMeTAD and the respective OPOTs single component HTM solution (15 mg in 0.5 mL of chlorobenzene without dopants). Finally, about 80 nm-thick silver was thermally evaporated onto the HTM layer at a pressure of 5×10^{-7} Torr in a metal-masked area of 0.04 cm^2 to complete the fabrication of the device with a structure of FTO/TiO_2/$CH_3NH_3PbI_3$/HTM/Ag.

References

1. Wang, L.; Chang, Bohong.; Li, Hui.; Wu, Y.; Zhang, L.; Yin, L. Electron Acceptor Molecule Doping Induced π-π Interaction to Promote Charge Transport Kinetics for Efficient and Stable 2D/3D Perovskite Solar Cells. *Angew. Chem. Int. Ed.* **2023**, *62*, e202304256.

2. Xiao, X.; Chu, Y.; Zhang, C.; Zhang, Z.; Qiu, Z.; Qiu, C.; Wang, H.; Mei, A.; Rong, Y.; Xu, G.; Hu, Y.; Han, H. Enhanced Perovskite Electronic Properties via A-site Cation Engineering. *Fundam. Res.* **2021**, *1*, 385-392.
3. Vo, T. H.; Shekhirev, M.; Kunkel, D. A.; Orange, F.; Guinel, M. J. F.; Enders, A.; Sinitskii, A. Bottom-up solution synthesis of narrow nitrogen-doped graphene nanoribbons. *Chem. Commun*. **2014**, *50*, 4172−4174.
4. Talele, H. R.; Chaudhary, A. R.; Patel, P. R.; Bedekar, A. V. Expeditious Synthesis of Helicenes Using an Improved Protocol of Photocyclodehydrogenation of Stilbenes. *ARKIVOC* **2011**, *2011*, 15-37.
5. Yang, K.; Liao, Q.; Huang, J.; Zhang, Z.; Su, M.; Chen, Z.; Wu, Z.; Wang, D.; Lai, Z.; Woo, H. Y.; Cao, Y.; Gao, P.; Guo, X. Intramolecular Noncovalent Interaction-Enabled Dopant-Free Hole- Transporting Materials for High-Performance Inverted Perovskite Solar Cells. *Angew. Chem., Int. Ed.* **2022**, *61*, e202113749.
6. Pal, S.; Fatma, K.; Ravichandran, V.; Dash, J. Triazolyl Dibenzo[a,c]phenazines Stabilize Telomeric G-quadruplex and Inhibit Telomerase. *Asian J. Org. Chem*. **2021**, *10*, 2921-2926.
7. Lin, Y.-S.; Abate, S.Y.; Lai, K.-W.; Chu, C.-W.; Lin, Y.-D.; Tao, Y.-T.; Sun, S.-S. New Helicene-Type Hole-Transporting Molecules for High-Performance and Durable Perovskite Solar Cells. *ACS Appl. Mater. Interfaces* **2018**, *10*, 41439−41449.
